# Atomically dispersed Iridium on Mo_2_C as an efficient and stable alkaline hydrogen oxidation reaction catalyst

**DOI:** 10.1038/s41467-024-48672-9

**Published:** 2024-05-18

**Authors:** Jinjie Fang, Haiyong Wang, Qian Dang, Hao Wang, Xingdong Wang, Jiajing Pei, Zhiyuan Xu, Chengjin Chen, Wei Zhu, Hui Li, Yushan Yan, Zhongbin Zhuang

**Affiliations:** 1 State Key Lab of Organic–Inorganic Composites and Beijing Advanced Innovation Center for Soft Matter Science and Engineering, Beijing University of Chemical Technology, Beijing, China; 2 Department of Chemical and Biomolecular Engineering, University of Delaware, Newark, DE USA; 3 Beijing Key Laboratory of Energy Environmental Catalysis, Beijing University of Chemical Technology, Beijing, China

**Keywords:** Fuel cells, Materials for energy and catalysis, Catalysis, Electrochemistry, Energy

## Abstract

Hydroxide exchange membrane fuel cells (HEMFCs) have the advantages of using cost-effective materials, but hindered by the sluggish anodic hydrogen oxidation reaction (HOR) kinetics. Here, we report an atomically dispersed Ir on Mo_2_C nanoparticles supported on carbon (Ir_SA_-Mo_2_C/C) as highly active and stable HOR catalysts. The specific exchange current density of Ir_SA_-Mo_2_C/C is 4.1 mA cm^−2^_ECSA_, which is 10 times that of Ir/C. Negligible decay is observed after 30,000-cycle accelerated stability test. Theoretical calculations suggest the high HOR activity is attributed to the unique Mo_2_C substrate, which makes the Ir sites with optimized H binding and also provides enhanced OH binding sites. By using a low loading (0.05 mg_Ir_ cm^−2^) of Ir_SA_-Mo_2_C/C as anode, the fabricated HEMFC can deliver a high peak power density of 1.64 W cm^−2^. This work illustrates that atomically dispersed precious metal on carbides may be a promising strategy for high performance HEMFCs.

## Abstract (web summary)

High-performance hydroxide exchange membrane fuel cells rely on the anode loading of platinum-group metals. Here, the authors report a highly active hydrogen oxidation electrocatalyst which contains atomically dispersed Ir on Mo2C nanoparticles supported on a carbon substrate.

## Introduction

Utilizing hydrogen energy presents a viable solution to reduce the dependence on fossil fuels and tackle environmental concerns^1,2^. In the pursuit of establishing hydrogen economy, fuel cells play crucial roles^3,4^. Hydroxide exchange membrane fuel cells (HEMFCs), which are analogs of proton exchange membrane fuel cells (PEMFCs) but using hydroxide exchange membranes, are promising due to their cost-effectiveness coming from the possibility to use platinum group metals (PGMs) free cathode electrocatalysts (e.g., Ag, Mn-Co oxides and Fe-N-C) and Ni-based bipolar plates^5,6^. However, high-performance anodes for HEMFCs still rely on PGMs^7^. Moreover, the pH effect on PGMs (e.g., Pt, Rh, Pd and Ir) leads to a significant slowdown of the hydrogen oxidation reaction (HOR) kinetics by approximately 2-3 orders of magnitude when changing the electrolyte from acid to alkaline, which results in high PGMs loadings on anode of HEMFCs^8^. Consequently, the anode cost in HEMFC substantially increases. Additionally, the poor stability of commonly used Pt-based catalysts necessitates higher catalyst loadings to enable a stable HEMFC, further increasing costs^9^. Hence, the development of highly efficient and stable catalysts with lower loading of PGMs for alkaline HOR is a pivotal target for the ongoing advancement of HEMFCs.

Significant efforts have been made to develop highly efficient catalysts for alkaline HOR^10^ and have shown that by tuning the microenvironment of active sites, the binding energies of HOR intermediates could be adjusted to improve the HOR kinetics^11^. Alloys, heterostructures and decorated catalysts have been reported with elevated HOR performance compared with the pristine metal catalysts by adjusting the H and OH binding energies^12–15^. Ir, which demonstrates enhanced OH bindings than Pt, has been shown to be a promising candidate for HOR in alkaline. Ir alloy (e.g., IrNi, IrMo and IrRu)^16–18^ and heterostructures (e.g., Ir/MoS_2_ and Ir/WO_x_)^19,20^ have been reported with enhanced HOR activities, even comparable to the state-of-the-art PtRu/C catalysts. The catalyst HOR stability is also strongly affected by the microenvironment of active sites^9,21^. Recent studies ascribed the poor stability of commercial carbon-supported Pt nanoparticle (NP) catalysts to the local carbon support corrosion^22,23^. Especially, PGMs can catalyze the carbon corrosion, making it especially serious at the interface of Pt NP and carbon support. Thus, a more stable substrate is desired to load the PGMs with less corrosion. Alternative supports, such as oxides, carbides, and nitrides, have been reported to improve the stability^21,24^. For example, TiO_2_-RuO_2_ was used as the support for Pt nanoparticles, and enhanced fuel cell stability was achieved^25^. Another method is to use a buffer to separate PGMs and the carbon substrates. For example, Dekel et. al.^26^ reported Pd/CeO_2_-C with enhanced stability, by using CeO_2_ as buffer substrate to load Pd nanoparticles, which could prevent the direct contact between Pd sites and the carbon support and thus mitigate the local carbon corrosion^27^. This work indicates that a rationally designed catalyst with a tailored microenvironment could also achieve high activity and stability.

Atomically dispersed metal catalysts show great potential for high-performance electrocatalysts due to their high atom-utilization and special microenvironment around the metal sites^28,29^. However, the commonly used M-N_4_-C type atomically dispersed catalysts do not show satisfactory performance for HOR, especially in HEMFC devices, because their significantly different metal microenvironments compared with those in pristine metal NPs may lead to improper binding to the HOR intermediates, such as adsorbed H (*H) and adsorbed OH (*OH)^30–32^. As a result, if a properly selected substrate can both host the atomically dispersed PGMs and achieve the optimized metal site microenvironment, it may simultaneously bring high HOR activity and stability.

Herein, we report an atomically dispersed Ir on Mo_2_C NPs supported on carbon (denoted as Ir_SA_-Mo_2_C/C) as a highly active and stable HOR catalyst in alkaline electrolyte. Mo_2_C NPs are used as the hosts for Ir atoms. The Mo_2_C shows Pt-like electronic structure, enabling the guest Ir to have a unique binding property with intermediates^33–35^. Mo_2_C also has high stability and strong interaction with PGMs, making Ir atoms atomically dispersed in the hexagonal Mo_2_C matrix. The obtained Ir_SA_-Mo_2_C/C catalyst achieves excellent activity for HOR, indicated by the high specific exchange current density of 4.1 mA cm^-2^_ECSA_ and mass activity at 50 mV versus reversible hydrogen electrode (RHE, the same hereafter) of 17.9 A mg^-1^_Ir_, which are 10 and 12 times that of Ir/C, and 2.6 and 4.3 times that of the current state-of-the-art PtRu/C, respectively. By using a low anode PGM loading of only 50 μg_Ir_ cm^-2^, the fabricated Ir_SA_-Mo_2_C/C HEMFC can deliver a high peak power density (PPD) of 1.64 W cm^-2^. The Ir_SA_-Mo_2_C/C illustrates high stability as well. It can work stably in a 120-h continuous operation and 30,000 cycles of accelerated durability test (ADT). Density functional theory (DFT) calculations reveal that the *d*-orbital of Ir_SA_-Mo_2_C is much different from that of the HOR inactive M-N_4_-C type atomically dispersed catalysts, and the high activity of Ir_SA_-Mo_2_C/C comes from the enhanced hydroxide binding as well as the optimized hydrogen binding. The present study demonstrates the advantages of the atomically dispersed metal on carbides in electrocatalysis.

## Results and Discussion

### Catalyst synthesis and characterization

**Fig. 1:**
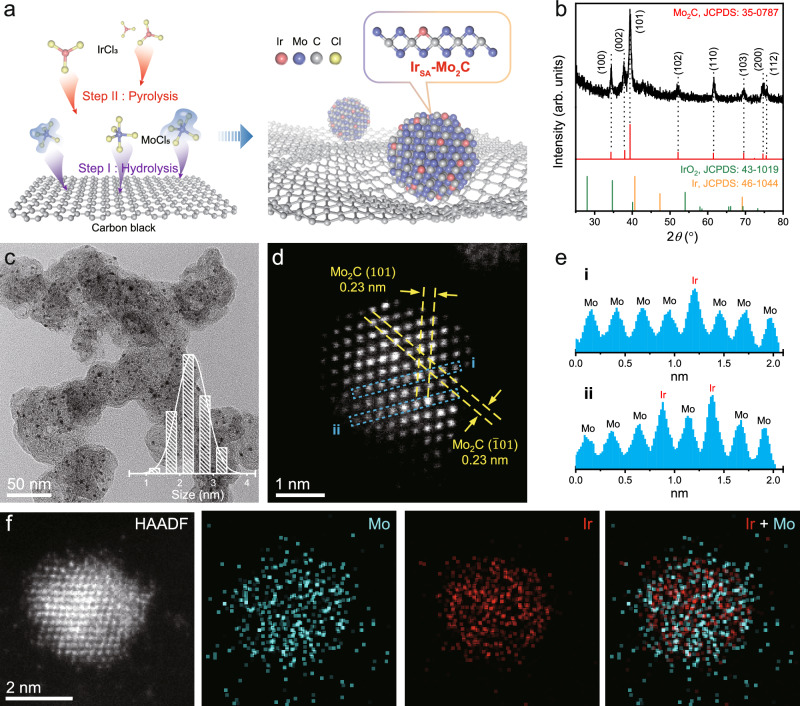
Synthesis of Ir_SA_-Mo_2_C/C and its characterizations. **a** Schematic illustration of the synthesis procedure. **b** PXRD pattern. **c** TEM image. The inset shows the particle size distribution. **d** AC-HAADF-STEM image. **e** Intensity profiles of the line i and ii in (**d**). **f** HAADF-EDS mapping results.

The Ir_SA_-Mo_2_C/C catalyst was synthesized by a two-step approach (Fig. 1a). Firstly, MoO_x_ was grown on pre-oxidized carbon black via the hydrolysis of MoCl_5_. Supplementary Fig. 1 shows the transmission electron microscopy (TEM) image and the corresponding energy-dispersive X-ray spectroscopy (EDS) mappings. It demonstrated that Mo was homogeneously dispersed on the carbon support. The broad shoulder peak in powder X-ray diffraction (PXRD) pattern (Supplementary Fig. 2) suggested the formation of amorphous MoO_x_. Subsequently, Ir was introduced by impregnation, followed by calcination at 900 °C under nitrogen environment. Under high temperature, carbon atoms would diffuse from carbon black to MoO_x_ and produce the Mo_2_C NPs^36^. At the same time, Ir was atomically dispersed on it to form the Ir_SA_-Mo_2_C/C catalyst because of the strong interaction between Ir atoms and Mo_2_C host.

Figure 1b shows the PXRD pattern of the Ir_SA_-Mo_2_C/C. All the diffraction peaks were assigned to hexagonal Mo_2_C without any signal from Ir-derived species, such as Ir and IrO_2_. The TEM image of Ir_SA_-Mo_2_C/C (Fig. 1c) clearly shows the well-dispersed NPs with an average size of 2.4 nm (inset of Fig. 1c) on the carbon supports. The aberration-corrected high-angle annular dark-field scanning TEM images (AC-HAADF-STEM, Fig. 1d) illustrated two sets of lattice with spacing of both 0.23 nm and angle of ca. 57°, which could be assigned to the (101) and ($\bar{1}01$) facets of hexagonal Mo_2_C^37^. Figure 1e exhibits the line intensity profile of the two areas in Fig. 1d. Isolated dots with particularly high intensity were observed, which was assigned to Ir atoms because of the higher atomic number of Ir than Mo^15^. It was confirmed that Ir substituted the Mo sites in Mo_2_C, and Ir were atomically isolated. Furthermore, the EDS mapping (Fig. 1f) verified the uniform dispersion of Ir and Mo. The Ir/Mo molar ratio in Ir_SA_-Mo_2_C/C was ca. 1:11 from EDS spectra (Supplementary Fig. 3), which was consistent with the ratio of ca. 1:10 tested by inductively coupled plasma optical emission spectra (ICP-OES). The Ir loading in Ir_SA_-Mo_2_C/C was ca. 3.1 wt% from ICP-OES.

**Fig. 2:**
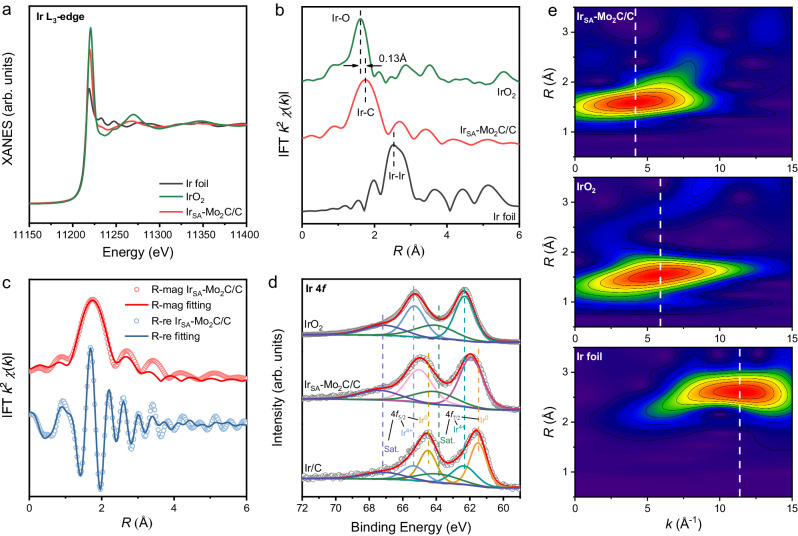
Structural analyzes of Ir_SA_-Mo_2_C/C. **a** Ir L_3_-edge XANES spectra. The spectra of IrO_2_ and Ir foil are shown as reference. **b** Corresponding FT *k*^2^-weighted EXAFS spectra. **c** FT-EXAFS fitting curves at *R*-space of Ir L_3_-edge. **d** Ir 4 *f* high resolution XPS spectra of Ir_SA_-Mo_2_C/C, Ir/C and IrO_2_. **e** Corresponding WT-EXAFS spectra of Ir_SA_-Mo_2_C/C, IrO_2_ and Ir foil.

Then, the electronic structures and coordination environments of Ir in Ir_SA_-Mo_2_C/C were further investigated by X-ray absorption spectroscopy (XAS). The Ir L_3_-edge X-ray absorption near-edge structure (XANES, Fig. 2a) of Ir_SA_-Mo_2_C/C exhibited the white line intensity between that of Ir foil and IrO_2_, revealing that the valence state of Ir in Ir_SA_-Mo_2_C/C was between that of Ir and IrO_2_^38^. The same conclusion was obtained from the Ir 4*f* X-ray photoelectron spectroscopy (XPS, Fig. 2d). The 4*f*_7/2_ peak of Ir/C could be deconvoluted into three peaks: Ir^0^ at 61.5 eV, Ir^4+^ at 62.4 eV and its satellite peak at 64.0 eV^39,40^. The existence of the Ir^4+^ species is likely from the surface oxidation of nanoparticles^41^. For IrO_2_, only the Ir^4+^ species and satellite peak were observed. The peak of Ir_SA_-Mo_2_C/C was located in between of those for Ir^0^ and Ir^4+^, indicting its medium oxidation state. Supplementary Fig. 4 shows the Mo 3*d* XPS spectra of Ir_SA_-Mo_2_C/C and Mo_2_C/C, indicating the proportion of Mo^6+^ species increased after introducing Ir. The Fourier-transformed *k*^2^-weighted extended X-ray absorption fine structure (EXAFS, Fig. 2b) shows that Ir_SA_-Mo_2_C/C exhibits a major peak at ca. 1.75 Å, which comes from the Ir−C path. No Ir−Ir path (2.52 Å, reference Ir foil) or Ir−O path (1.62 Å, reference IrO_2_) was observed. We further performed the wavelet transform EXAFS (Fig. 2e) analysis to distinguish back-scattering atoms in the k-space. The Ir_SA_-Mo_2_C/C revealed only one intensity maximum located at 4.1 Å^−1^, which was different from that of IrO_2_ (5.8 Å^−1^) and Ir foil (11.4 Å^−1^), suggesting the unique Ir−C scattering path in Ir_SA_-Mo_2_C/C^31^. Furthermore, the FT-EXAFS fitting in the *R*-space (Fig. 2c) and the *k*-space (Supplementary Fig. 5) suggested the Ir−C scattering path of 2.10 Å after phase correction with the coordination number of ca. 3.1, which was reasonably close to the Mo−C path in Mo_2_C located at 2.11 Å with corresponding coordination number of 3. The fitting curves for Ir foil and IrO_2_ were shown in Supplementary Fig. 6, and results were summarized in Supplementary Table 1. Thus, the structure of Ir_SA_-Mo_2_C was confirmed as the Ir atoms partially replaced the Mo atoms in the hexagonal Mo_2_C lattice and thus coordinated with C.

### High HOR activity of the Ir_SA_-Mo_2_C/C

**Fig. 3:**
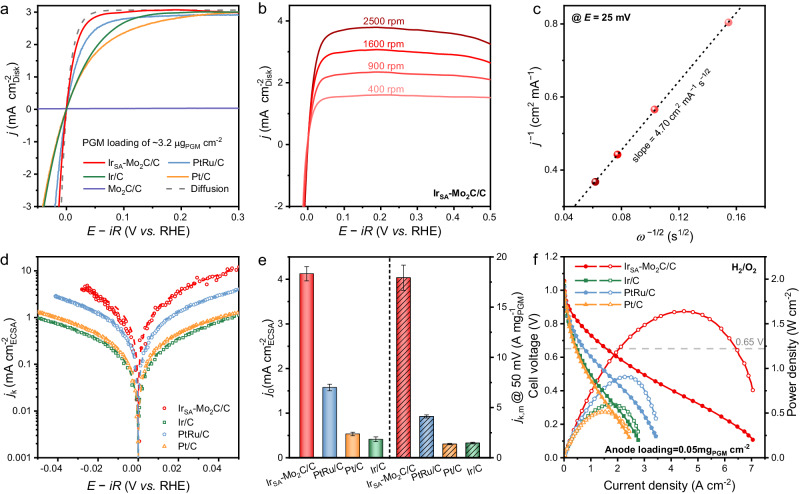
HOR performance. **a** Polarization curves of the catalysts in H_2_-saturated 0.1 M KOH solution with the rotation rate of 1600 rpm and the scan rate of 5 mV s^−1^. The catalyst loading was 3.2 μg_PGM_ cm^−2^ for Ir_SA_-Mo_2_C/C, Ir/C, Pt/C and PtRu/C. The potentials were *iR* corrected and the *R* values for Ir_SA_-Mo_2_C/C, Ir/C, Pt/C and PtRu/C measurements were 36.1 ± 0.10, 35.9 ± 0.12, 36.2 ± 0.10 and 36.0 ± 0.07 Ω, respectively. **b** Polarization curves of Ir_SA_-Mo_2_C/C at different rotation rates. **c** The corresponding Koutecky-Levich plot at 25 mV. **d** Tafel plots of the catalysts where the current densities were normalized to their ECSA. The dash line shows the Butler-Volmer fitting curves. **e** The specific exchange current density (*j*_0,ECSA_) and the mass normalized kinetic current density (*j*_k,m_) at 50 mV. Error bars are s.d. of at least three sets of experimental repeats. **f** H_2_/O_2_ HEMFC performances using different anode catalysts. The anode PGM loadings were controlled as 0.05 mg_PGM_ cm^−2^. The cathode for all the HEMFCs was 0.4 mg_Pt_ cm^−2^ Pt/C. The cell, anode and cathode humidifier temperatures were 95, 92 and 95 °C, respectively. The anode and cathode were flowed with 1.0 L min^−1^ of H_2_ and 0.5 L min^−1^ of O_2_, respectively. The backpressures were 250 kPag for both sides.

The HOR electrocatalytic activity of the Ir_SA_-Mo_2_C/C was firstly investigated by the rotating disk electrode (RDE) method in H_2_-saturated 0.1 M KOH using the standard three-electrode system. The commercial PtRu/C, Pt/C and IrO_2_ were tested at the same condition for comparison. The commonly used carbon-based Ir single atom catalysts (i.e., Ir_SA_-N_4_-C, synthesized through the procedure reported in refs. ^32,42^. Ir/C (synthetic procedure shown in Methods, TEM images and XRD pattern shown in Supplementary Fig. 7), and Ir nanocluster on Mo_2_C supported on carbon (Ir_NC_-Mo_2_C/C, synthetic procedure shown in Methods, TEM images and XRD pattern shown in Supplementary Fig. 8) were tested as control samples. Different from the negligible HOR activity of the Ir_SA_-N_4_-C (Supplementary Fig. 9, similar performance was reported by Q. Wang et al.)^43^ the Ir_SA_-Mo_2_C/C illustrated high HOR performance, indicated by the steeply increased anodic current density (Fig. 3a). The Mo_2_C/C support showed scarce HOR activity, suggesting that the high performance of Ir_SA_-Mo_2_C/C came from the Ir sites. And the Ir_SA_-Mo_2_C/C also displayed higher HOR activity than Ir_NC_-Mo_2_C/C, indicating the benefit of the monoatomic dispersed Ir in Mo_2_C. The Ir_SA_-Mo_2_C/C showed better performance than the other Ir-based catalysts, such as Ir/C and IrO_2_ (Fig. 3a and Supplementary Fig. 10), as well as the commonly used Pt-based catalysts, such as Pt/C and the state-of-the-art PtRu/C (Fig. 3a).

We further tested the polarization curves of Ir_SA_-Mo_2_C/C at different rotating speeds (Fig. 3b). By fitting with the Koutecky-Levich equation (Fig. 3c), a slope of 4.70 cm^2^ mA^−1^ s^−1/2^ was obtained, which was reasonably close to the theoretical value of the 2e HOR process (4.87 cm^2^ mA^−1^ s^−1/2^) and suggested the anode current mainly derived from HOR^44,45^.

In order to quantitatively compare the HOR activity, we calculate the specific exchange current density (*j*_0,ECSA_) based on the electrochemical surface area (ECSA) and the mass activity at 0.05 V of the catalysts. The ECSA of the catalysts are determined by CO stripping voltammetry, and the same specific charge of 420 μC cm^−2^ is used for all the catalysts (Supplementary Fig. 11 and Supplementary Table 2). The Ir_SA_-Mo_2_C/C exhibits an ECSA of 156 m^2^ g^−1^_Ir_, which was larger than the ECSA values of Ir/C (128 m^2^ g^−1^_Ir_), Pt/C (97 m^2^ g^−1^_Pt_) and PtRu/C (107 m^2^ g^−1^_PtRu_)^46^. The high ECSA was benefited from the atomically dispersion of Ir in Ir_SA_-Mo_2_C/C, which brought high utilization of Ir. Figure 3d shows the ECSA normalized Tafel plots. By fitting with the Butler-Volmer equation according to Tafel-Volmer pathway^45^, the Ir_SA_-Mo_2_C/C provides the greatest *j*_0,ECSA_ of 4.1 ± 0.16 mA cm^−2^_ECSA_ (Fig. 3e), which was 2.6, 7.7 and 10 times as high as that for PtRu/C (1.6 ± 0.07 mA cm^−2^_ECSA_), Pt/C (0.53 ± 0.041 mA cm^−2^_ECSA_) and Ir/C (0.41 ± 0.054 mA cm^−2^_ECSA_), respectively. The measured specific *j*_0,ECSA_ of PtRu/C, Pt/C and Ir/C reproduced the literature data^13,45,47^, implying a reliable evaluation of HOR activity. The Ir_SA_-Mo_2_C/C also exhibited a high mass activity at 50 mV of 17.9 ± 1.2 A mg^−1^_Ir_, which was 4.3, 13 and 12 times higher than that of PtRu/C (4.12 ± 0.16 A mg^−1^_PtRu_), Pt/C (1.37 ± 0.06 A mg^−1^_Pt_) and Ir/C (1.47 ± 0.08 A mg^−1^_Ir_), respectively. The more significant improvement of the mass activity is attributed to the higher Ir utilization of Ir_SA_-Mo_2_C/C. Ir_SA_-Mo_2_C/C also showed great advantages in both specific activity and mass activity compared with the previously reported high-performance HOR catalysts (summarized in Supplementary Fig. 12 and Supplementary Table 3).

Encouraged by the superior HOR activity of the obtained Ir_SA_-Mo_2_C/C catalyst, we employed it as the anode catalyst to assemble the HEMFCs. The membrane electrode assembly (MEA) was made by using Ir_SA_-Mo_2_C/C as anode catalyst with a low PGM loading of 0.05 mg_Ir_ cm^−2^ (confirmed by ICP-OES), commercial Pt/C (0.4 mg_Pt_ cm^-2^) as cathode catalyst and the PiperION™ A15 (Versogen) as the hydroxide exchange membrane. The control MEAs were fabricated by only replacing the anode catalyst to PtRu/C, Pt/C and Ir/C with the same loading of 0.05 mg_PGM_ cm^−2^. Figure 3f displays the polarization and power density curves of the as-prepared HEMFCs under H_2_/O_2_ condition. The Ir_SA_-Mo_2_C/C HEMFC exhibited excellent performance, which could deliver high PPD of 1.64 W cm^−2^, and is much better than the HEMFCs using PtRu/C (0.91 W cm^−2^), Pt/C (0.51 W cm^−2^) and Ir/C (0.60 W cm^−2^). The Ir_SA_-Mo_2_C/C HEMFC achieved a high current density of 1.60 A cm^−2^ at the cell voltage of 0.65 V, which was also higher than that of PtRu/C HEMFC (0.76 A cm^−2^), Pt/C HEMFC (0.43 A cm^−2^) and Ir/C HEMFC (0.49 A cm^-2^). The Ir_SA_-Mo_2_C/C HEMFC also showed high performance under H_2_/air(CO_2_-free) condition, which delivered a PPD of 0.90 W cm^−2^ and reached a current density of 1.11 A cm^−2^ at 0.65 V (Supplementary Fig. 13). Furthermore, we normalized the PPD of these MEAs to the anode catalyst loading to calculate the anode mass activity. As shown in Supplementary Fig. 14 and Supplementary Table 4, Ir_SA_-Mo_2_C/C HEMFC delivered a high anode mass activity of 32.8 W mg^−1^_Ir_, which was superior to the previously reported HEMFCs.

### Enhanced HOR stability of Ir_SA_-Mo_2_C/C

**Fig. 4:**
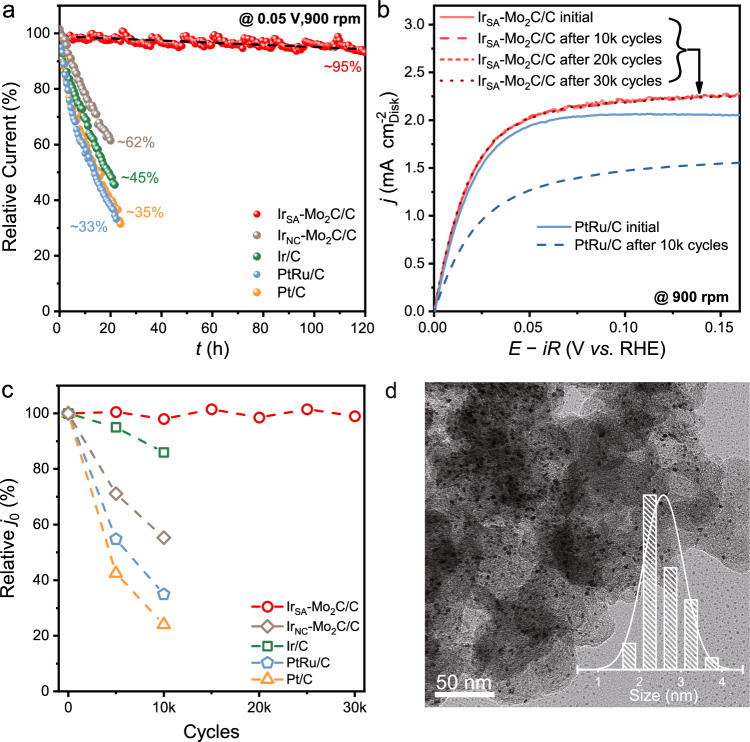
Stability evaluation. **a** The chronoamperometry at 50 mV of Ir_SA_-Mo_2_C/C, Ir_NC_-Mo_2_C/C, Ir/C, Pt/C and PtRu/C tested in H_2_-saturated 0.1 M KOH with a rotation rate of 900 rpm. **b** polarization curves for Ir_SA_-Mo_2_C/C and PtRu/C before and after ADT. The scan rate was 2 mV s^−1^ and the rotation rate was 900 rpm. The potentials were *iR* corrected and the *R* values for Ir_SA_-Mo_2_C/C and PtRu/C measurements were 37.0 ± 0.08 and 37.6 ± 0.10 Ω, respectively. **c** The relative exchange current density evolution in the ADT. **d** TEM image of the Ir_SA_-Mo_2_C/C after 30,000 cycles of ADT. The inset shows particle size distribution.

The HOR stability of Ir_SA_-Mo_2_C/C was evaluated by RDE method. Two techniques, chronoamperometry and cyclic voltammetry for ADT, were carried out. Figure 4a exhibits the chronoamperometry response of Ir_SA_-Mo_2_C/C and the control catalysts in H_2_-saturated 0.1 M KOH at a constant potential of 50 mV. The current density of Ir_SA_-Mo_2_C/C maintained ca. 95% of the initial value after continuously operating for 120 h. The polarization curves before and after 120 h operation (Supplementary Fig. 15) showed negligible decay. While for the Ir_NC_-Mo_2_C/C, Ir/C, Pt/C and PtRu/C, the current density remained only 62 %, 45 %, 35 % and 33 % after a short test of 20 h, respectively. The decay rate of Ir_SA_-Mo_2_C/C was 0.042 % h^−1^, much smaller than those of Ir_NC_-Mo_2_C/C (1.9 % h^−1^), Ir/C (2.5 % h^−1^), Pt/C (2.9 % h^−1^) and PtRu/C (3.0 % h^−1^), showing nearly two order of magnitude slowdown of the catalyst decay. Supplementary Fig. 16 and Supplementary Table 5 summarize the decay rate of Ir_SA_-Mo_2_C/C and the reported HOR catalysts, where Ir_SA_-Mo_2_C/C showed great advantage.

The stable HOR performance of Ir_SA_-Mo_2_C/C was also determined by ADT. After scanning at a potential window of 0 ~ 0.5 V with the scanning rate of 0.1 V s^−1^ for 30,000 cycles, Ir_SA_-Mo_2_C/C did not show any decline (Fig. 4b). By comparison, PtRu/C, Pt/C, Ir/C and Ir_NC_-Mo_2_C/C exhibited dramatic decline in kinetic current (Fig. 4b and Supplementary Fig. 17). To quantitatively compare the activity loss in ADT, the corresponding exchange currents of these catalysts were summarized in Fig. 4c. Ir_SA_-Mo_2_C/C maintained its activity in the whole process of ADT without any decline, while PtRu/C, Pt/C, Ir/C and Ir_SA_-Mo_2_C/C suffered from a decay of 65 %, 76 %,14 % and 45 % in *j*_0_ after scanning for 10,000 cycles, respectively. The TEM image of Ir_SA_-Mo_2_C/C after ADT (Fig. 4d) showed the same structure with almost the same size distribution of NPs (ca. 2.5 nm) compared with the fresh catalyst. The HRTEM image (Supplementary Fig. 18) exhibited lattice spacing of 0.23 nm belonging to the Mo_2_C (101). The EDS mapping shows the homogenous dispersion of Ir and Mo atoms in the NPs after ADT. Overall, these results indicated the good stability and the Ir_SA_-Mo_2_C/C maintained its microstructure during HOR process.

The Ir_SA_-Mo_2_C/C was also stable under the HEMFC working conditions. Supplementary Fig. 19 shows the galvanostatic discharge curve at 500 mA cm^−2^ at 80 °C of the Ir_SA_-Mo_2_C/C HEMFC under H_2_/O_2_ condition. It showed negligible voltage loss after 50 h of test.

### Mechanism investigation

**Fig. 5:**
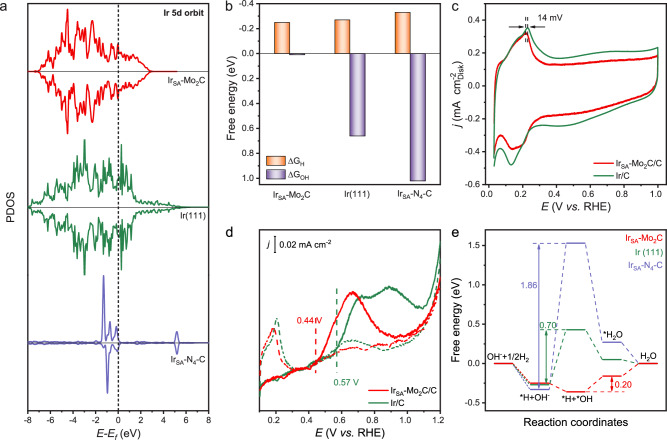
Mechanism investigation. **a** The PDOS diagram for *d* orbital of metals in Ir_SA_-Mo_2_C, Ir (111) and Ir_SA_-N_4_-C. **b** The calculated adsorption Gibbs free energy of H and OH. **c** CV curves of Ir-Mo_­2_C/C and Ir/C obtained in Ar-saturated 0.1 M KOH without *iR*-correction. The scan rate is 20 mV s^−1^. **d** CO-stripping curves of Ir_SA_-Mo_2_C/C and Ir/C obtained in 0.1 M KOH without *iR*-correction. The scan rate was 10 mV s^−1^. The solid lines are CO-stripping voltammetry. The dash lines are CV curves after the CO-stripping. **e** The Gibbs free energy diagrams of HOR on the Ir_SA_-Mo_2_C, Ir (111) and Ir_SA_-N_4_-C.

Based on the DFT calculations, electronic structures and reaction pathways of Mo_2_C (101) slab model with 10% of Mo atoms substituted by Ir (model shown in Supplementary Fig. 20 and cell parameter shown in Supplementary Table 6) were studied to deeply understand the origin of the high HOR activity of atomically dispersed Ir_SA_-Mo_2_C/C catalyst at the atomistic level. The Ir (111) slab and the typical carbon-based Ir_SA_-N_4_-C single-atom catalyst (model shown in Supplementary Fig. 21) were also studied for comparison. Bader charge analysis (Supplementary Fig. 22) suggests the electron transfer from surface Mo to the adjacent Ir in Ir_SA_-Mo_2_C, consistent with the XPS results.The partial density of states (PDOS) plots (Fig. 5a) show that the Ir-5*d* orbitals of Ir_SA_-N_4_-C are very localized. By contrast, the Ir-5*d* orbitals of the Ir_SA_-Mo_2_C exhibit a large degree of delocalization, suggesting its metal-like electronic structure of Ir atoms in Ir_SA_-Mo_2_C, which is similar to the 5*d* bands of Ir (111). Such maintained metallicity of Ir in Ir_SA_-Mo_2_C was benefited from the Pt-like electronic structure of the Mo_2_C substrate, which makes the dispersed Ir sites behave more like single atom alloy and thus brings high HOR activity similar to Ir (111). Actually, it is noteworthy that almost all reported high-efficient HOR catalysts rely on the metallic sites^48^. Furthermore, the total density of states (DOS, Supplementary Fig. 23) of Ir_SA_-Mo_2_C exhibits a higher occupation (with higher carrier concentration) at the Fermi level compared with Ir_SA_-N_4_-C, suggesting a much better electron conductivity^49^.

The adsorption energies of the HOR intermediates control the activity of Ir_SA_-Mo_2_C. Although the mechanism of alkaline HOR was still under debate, H and OH adsorptions were generally considered as the key step in the kinetics of alkaline hydrogen oxidation^50–52^. Figure 5b summarizes the calculated adsorption Gibbs free energy (∆*G*) of adsorbed H, OH. The ∆*G*_H_ value is considered as the primary descriptor for the HOR kinetics. The H adsorptions on the different sites of Ir_SA_-Mo_2_C were evaluated (Supplementary Fig. 24 and Supplementary Table 7), and it was found that the Ir-Mo hollow sites offer the suitable ∆*G*_H_ of −0.25 eV. The Ir_SA_-Mo_2_C illustrated slightly weaker H binding than Ir (111) surface (∆*G*_H_ = − 0.27 eV), which was more optimized for the HOR process^53^. The slightly weakened H binding was indicated by the 14 mV of negatively shift of the H_upd_ peak as well (Fig. 5c). When an Ir cluster was placed on the Mo_2_C (101) substrate (Ir_NC_-Mo_2_C, model shown in Supplementary Fig. 25), a stronger H adsorption with ∆*G*_H_ = − 0.40 eV was found. Pure IrO_2_ (110) (∆*G*_H_ = − 0.64 eV) and Mo_2_C (101) (∆*G*_H_ = − 0.57 eV) also exhibited the too strong H bindings (shown in Supplementary Fig. 26–27), leading to the poorer HOR activity, which were consistent to the experimental results. In addition, although Ir_SA_-N_4_-C demonstrated a stronger H binding (∆*G*_H_ = − 0.33 eV) than Ir (111) surface, it did not remarkably deviate from the optimal value of H adsorption energy.

Besides ∆*G*_H_, the free energy of OH adsorption (∆*G*_OH_) is considered as another important indicator for activity in alkaline HOR. Markovic et al. considered that the *OH combined with *H to generate water through the bifunctional mechanism, and thus accelerating the H desorption^11^. While Chen et al. proposed stronger H-bond network among interfacial water molecules, which was important for the H transfer^54^. Both possible mechanisms suggested that the enhanced OH adsorption could improve HOR. Here, the Ir_SA_-Mo_2_C, Ir and Ir_SA_-N_4_-C catalysts illustrated much different values of ∆*G*_OH_ (Fig. 5b). Based on the screening of the different sites of Ir_SA_-Mo_2_C, it was found that the Mo sites were more favored to adsorb OH (Supplementary Fig. 28 and Supplementary Table 6). Compared with Ir (111) surface (∆*G*_OH_ = 0.66 eV), Ir_SA_-Mo_2_C (∆*G*_OH_ = 0.01 eV) showed dramatic enhancement of OH adsorption, while Ir_SA_-N_4_-C (∆*G*_OH_ = 1.02 eV) possessed an increased ∆*G*_OH_ value. The enhanced OH adsorption of Ir_SA_-Mo_2_C could be concluded by the lower onset potential of Ir_SA_-Mo_2_C/C than Ir/C in the CO-stripping voltammetry (Fig. 5d). The much weaker OH adsorption of the Ir_SA_-N_4_-C catalyst should be the origin of its poor HOR performance.

Therefore, the calculated adsorption-free energies suggest the high HOR performance of the Ir_SA_-Mo_2_C/C can be attributed to its optimized H binding and enhanced OH binding. The Gibbs free energy profiles of the HOR process on each catalyst are displayed in Fig. 5e. Due to the weak OH adsorption, the potential-determining steps on Ir (111) and Ir_SA_-N_4_-C were both the OH^−^ adsorption step (i.e., *H + * + OH^−^ →*H + *OH + e^−^), and the energy barriers were 0.70 and 1.86 eV, respectively. Four reaction paths were considered on Ir_SA_-Mo_2_C through different adsorption sites (Supplementary Fig. 29). Because of the enhanced OH adsorption on Mo sites, all the paths exhibited lower energy barrier for OH^−^ adsorption step, giving the potential determining step of water generation step (i.e., *H + *OH → *H_2_O) on Ir_SA_-Mo_2_C. The favorite path showed a lower energy barrier of 0.20 eV, indicating its fastest HOR kinetics. The Gibbs free energy profiles for Ir_NC_-Mo_2_C, Mo_2_C (101) and IrO_2_ (110) were calculated as well (Supplementary Fig. 25–27), which showed higher energy barriers of 0.80, 1.57, and 1.03 eV, respectively.

In summary, we have developed an atomically dispersed Ir (i.e., Ir_SA_-Mo_2_C/C) as a highly efficient and stable HOR catalyst in alkaline electrolyte to achieve high HEMFC performance using low anode PGM loading. The unique Mo_2_C substrate properly adjusts the Ir sites to achieve optimized H binding and enhanced OH binding, and thus significantly promotes the HOR kinetics on Ir_SA_-Mo_2_C/C. The Ir_SA_-Mo_2_C/C exhibits a high mass activity of 17.9 A mg_Ir_^−1^ at 0.05 V, surpassing the state-of-the-art PtRu/C by 4.3 times. The high stability of the Mo_2_C substrate also promotes the durability of the Ir_SA_-Mo_2_C/C, exhibiting a decay rate 72 times smaller than the PtRu/C. This work highlights that carbides can serve as an excellent substrate to host the atomically dispersed PGMs, and this type catalyst is promising in reducing the PGM requirement in electrocatalysis.

## Methods

### Materials

MoCl_5_ (99.99%), Ethanol (99.5%), HNO_3_ (65%) and KOH (99.999%) was purchased from Aladdin. IrCl_3_ · xH_2_O (99.9%) was obtained from Sigma Aldrich. Ketjen black EC-300J was purchased from Lion Corporation. Nafion solution, Pt/C (20%, Johnson Matthey), PtRu/C (40% Pt and 20% Ru, Johnson Matthey), IrO_2_ and gas diffusion layers (SGL 29BC) were obtained from Suzhou Sinero Technology Co., Ltd. The hydroxide exchange membrane (PiperION™ A15) and the ionomer were bought from Versogen. Ultrapure water (18.2 MΩ·cm, Mili-Q, Merck) was used in the synthesis of catalysts and electrochemical measurements.

### Syntheses of the catalysts

#### Synthesis of the oxidized-carbon black

1 g of Ketjen black (EC-300J) was dispersed in 100 mL of 65% HNO_3_ solution through ultrasonication for 15 min. The mixture was then heated to 85 °C with vigorous stirring at refluxed state for 1 h. Then it was centrifuged and dispersed in water, followed by filtering and washing with plenty of water. Finally, the filter cake was dried by lyophilization.

#### Synthesis of the Ir_SA_-Mo_2_C/C catalyst

Firstly, 200 mg of the as-prepared oxidized-carbon black and 380 mg of MoCl_5_ were dispersed in 30 mL of ethanol through ultrasonication for 15 min, followed by adding 5 mL of water. Then, the mixture was heat to 80 °C with vigorous stirring for 8 h. The product was centrifuged and dispersed in water, followed by filtering and washing with plenty of water. The MoO_x_/C was obtained by lyophilization. Secondly, 0.03 mM of IrCl_3_ · xH_2_O (12 mg) and 109 mg of the obtained MoO_x_/C were dispersed in 50 mL of water by ultrasonication for 1 h. The solvent was then evaporated using rotary evaporator at 50 °C. The residue was collected and dried overnight. Then, it was grinded and calcined at 900 °C under N_2_ atmosphere for 1 h to obtain Ir_SA_-Mo_2_C/C catalyst. The Ir loading in the catalyst was ca. 3.1% determined by ICP-OES.

#### Synthesis of the Ir_NC_-Mo_2_C/C catalyst

Ir_NC_-Mo_2_C/C catalyst was prepared by the same method as Ir_SA_-Mo_2_C/C catalyst, except using 3 times the amounts of IrCl_3_ · xH_2_O.

#### Synthesis of the Ir/C catalyst

0.03 mM of IrCl_3_ · xH_2_O (12 mg) and 109 mg of oxidized-carbon black were dispersed in 50 mL of water by ultrasonication for 1 h. The solvent was then evaporated using rotary evaporator at 50 °C. The residue was collected and dried overnight. Then, it was grinded and calcined at 900 °C under N_2_ atmosphere for 1 h to obtain Ir/C catalyst.

#### Synthesis of the Ir_SA_-N_4_-C catalyst

The synthesis of Ir_SA_-N_4_-C was followed by the procedure reported by Li et al.^42^. Typically, 2 mmol of Zn(NO_3_)_2_·6H_2_O (594 mg) and 0.2 mmol of Ir(acac)_3_ (98 mg) were simultaneously dissolved in 7.5 mL of methanol to form a uniform solution. Another solution was made by dissolving 8 mmol of 2-methyl imidazole (656 mg) to 15 mL of methanol. After rapidly mixing the two solutions and vigorously stirring for 5 min, the mixed solution was transferred into a 50 mL Telfon-lined stainless-steel autoclave. The sealed vessel was heated at 120 °C for 4 h before it was cooled to room temperature. Then it was separated by centrifugation, washed with methanol for four times, and dried under vacuum for 8 h. Finally, it was put into a tube furnace and pyrolyzed at 900 °C for 3 h under Ar atmosphere to obtain Ir_SA_-N_4_-C catalyst.

### Characterizations

Powder X-ray powder diffraction (PXRD) patterns were recorded on a Rigaku D/Max 2500 VB2 + /PC X-ray powder diffractometer equipped with Cu K_α_ radiation (λ = 0.154 nm). Transmission electron microscopy (TEM) images were obtained on a HITACHI HT7700 transmission electron microscope operating at 100 kV. Aberration-corrected high-angle annular dark-field scanning transmission electron microscopy (AC-HAADF-STEM) and energy dispersive X-ray spectrometry (EDS) elemental mapping were performed using FEI TITAN 80-300 operating at 300 kV. The X-ray photoelectron spectra (XPS) were measured using a Thermo Fisher ESCALAB 250Xi XPS system with a monochromatic Al K_α_ X-ray source. All binding energies were calibrated to the C 1 s peak (284.8 eV). Inductively coupled plasma optical emission spectroscopy (ICP-OES, Optima 7300 DV, Perkin Elmer) was used to determine the metal contents.

### XAS measurements

X-ray absorption spectroscopy (XAS) at the Ir L_3_-edge (11215 eV) were measured at 1W1B station in Beijing Synchrotron Radiation Facility (BSRF, operated at 2.5 GeV with a maximum current of 250 mA). The XAS data of the samples were collected at room temperature in fluorescence excitation mode using a Lytle detector. All the samples were mixed with graphite and ground uniformly and then pressed into a 10 mm plate with a thickness of 1 mm.

The EXAFS data were processed by Athena and Artemis in the IFEFFIT packages. Firstly, the EXAFS spectra were obtained by Fourier transforming the $\chi \left(k\right)$ data with Hanning windows (d*k* = 0.5 Å^−1^) to separate the contributions from different coordination shells. Then, quantitative structural parameters around the Ru atom were obtained by fitting with the following EXAFS equation using the Artemis module.

1 $$\chi \left(k\right)={\sum}_{j}\frac{{N}_{j}{S}_{0}^{2}{F}_{j}\left(k\right)}{k{R}_{j}^{2}}\exp \left[-2{k}^{2}{\sigma }_{j}^{2}\right]\exp \left[-\frac{2{R}_{j}}{\lambda \left(k\right)}\right]\sin \left[2k{R}_{j}+{\phi }_{j}\left(k\right)\right]$$

where ${S}_{0}^{2}$ is the amplitude reduction factor, ${F}_{j}\left(k\right)$ is the effective curved-wave backscattering amplitude, ${N}_{j}$ is the number of neighbor atoms from different coordination shells, ${R}_{j}$ is the distance between the core atom and neighbor atoms, $\lambda \left(k\right)$ is the mean free path in Å, ${{\phi }_{j}}(k)$ is the phase shift (including the phase shift for each shell and the total central atom phase shift), ${\sigma }_{j}^{2}$ is the Debye-Waller parameter of the different atomic shells. The functions ${F}_{j}\left(k\right)$, $\lambda \left(k\right)$ and ${\phi }_{j}\left(k\right)$ were calculated by the ab initio code FEFF 8.2. During the fitting process, ${S}_{0}^{2}$ was fixed, while R, ${\sigma }^{2}$ and the edge-energy shift $\Delta {E}_{0}$ were allowed to run freely.

### Electrochemical measurements

The electrochemical measurements were operated at room temperature in a standard three-electrode electrochemical system controlled by a potentialstat (V3, Princeton Applied Research). A homemade glass electrochemical cell equipped with three-electrode assembly was used. The cell and glassware were cleaned by 3:1 mixture of concentrated sulfuric acid and hydrogen peroxide (30%), rinsed thoroughly and boiled with ultrapure water before measurements. A catalyst thin film on glassy carbon (GC) rotating disk electrode RDE (5 mm in diameter, Pine Research Instrumentation) was used as the working electrode. The surface of GC was polished with 0.05 mm Al_2_O_3_ powder and cleaned with water under ultrasonic. The catalyst ink was made by ultrasonically dispersing 1 mg of as-prepared Ir_SA_-Mo_2_C/C catalyst in a mixed solution containing 250 μL of distilled water, 750 μL of ethanol, and 5 μL of 5 wt % Nafion. Then 20 μL of the catalyst ink was transferred onto the GC substrate to yield a thin film electrode. The Ir loading was ca. 3.2 μg_Ir_ cm^−2^ according to the result of ICP-OES. The reversible hydrogen electrode (RHE, Phychemi Co., Ltd.) and a Pt wire (99.999%, Gaoss Union Co., Ltd.) were used as the reference and counter electrodes, respectively. The fresh 0.1 M KOH solution with the pH of ca. 12.8 ± 0.1, prepared by dissolving 2.81 g of KOH in 500 mL of water, was used as the electrolyte for all RDE measurements.

Cyclic voltammetry (CV) was carried out to analyze the electrochemical properties of the catalysts. The recorded potentials were *iR*-corrected. The solution resistance (*R*) was measured using AC-impendence from 200 kHz to 100 mHz with a voltage perturbation of 5 mV. The HOR activity was calculated using a positive-going direction polarization curves obtained in a H_2_-saturated 0.1 M KOH solution with the rotation rate of 1600 rpm and the scanning rate of 5 mV s^−1^. For the stability test, the catalyst loading was controlled at ca. 7 μg_Ir_ cm^−2^. A homemade fluorinated ethylene propylene electrochemical cell equipped was used. Two techniques were used to evaluated the stability. The rotation rate was change to 900 rpm to reduce the mechanical detachment of catalyst from the surface of RDE. Firstly, the chronoamperpmentry at a constant potential of 0.05 V was used. Secondly, the CV cycling was used for the accelerated durability test (ADT) by scanning the potential between 0 and 0.5 V with the scanning rate of 100 mV s^−1^. To eliminate the influence of CO_2_ from air, the electrolyte was refreshed each 50 h.

### Evaluation of activity and ECSA

The HOR/HER activity was represented by the exchange current density (*j*_0_), which was obtained by fitting kinetic current (*i*_k_) with the Butler-Volmer equation:

2 $${i}_{k}={j}_{0}{A}_{{{{\rm{s}}}}}\left[\exp \left(\frac{{\alpha }_{{{{\rm{a}}}}}F\eta }{{RT}}\right)-\exp \left(\frac{-{\alpha }_{{{{\rm{c}}}}}F\eta }{{RT}}\right)\right]$$

where *α*_a_ and *α*_c_ are the transfer coefficients for HOR and HER, respectively, with *α*_a_ + *α*_c_ = 1 (Tafel-Volmer pathway). *A*_s_ is the electrochemical surface area (ECSA), and *η* is the overpotential. *i*_k_ is calculated by the Koutecky-Levich equation:

3 $$\frac{1}{i}=\frac{1}{{i}_{k}}+\frac{1}{{i}_{d}}$$

where *i* is the measured current, *i*_k_ is the kinetic current, and *i*_d_ is the diffusion limited current. *i*_d_ is defined from the following equation:

4 $${i}_{d}={i}_{l}\left[1-\exp \left(-\frac{2F\eta }{{RT}}\right)\right]$$

where *η* is the overpotential and *i*_l_ is the maximum current obtained from polarization curves.

The ECSA was evaluated by CO-stripping voltammetry. A monolayer of CO was firstly adsorbed onto the catalyst’s surface by holding at 0.1 V for 10 min. The electrolyte was then flushed with Ar for 10 min to remove dissolved CO completely. Then, the adsorbed CO was stripped by scanning between 0.1 and 1.2 V at a scan rate of 10 mV·s^−1^.

### HEMFCs fabrications and tests

The synthesized Ir_SA_-Mo_2_C/C or the control catalysts were used as the anode catalyst with the loading of 0.05 mg_PGM_ cm^−2^. Pt/C (HiSpec 4000, 40 wt% Pt, Alfa Aesar) was used as the cathode catalyst with the loading of 0.4 mg_Pt_ cm^−2^. The catalyst ink was sprayed onto both sides of PiperION™ A15 membrane (17 µm) to fabricate a catalyst coated membrane (CCM) with the electrode area of 5 cm^−2^. All CCMs were immersed into 3 M NaOH solution overnight and then rinsed thoroughly with deionized water to remove all excess NaOH. The rinsed CCM was assembled with a fluorinated ethylene propylene (FEP) gasket, a GDL (SGL 29 BC), a graphite bipolar plate with 5 cm^2^ flow field and a metal current collector for each side to complete the full HEMFC. Fuel cell test system (Scribner 850 g) equipped with a backpressure module was used to evaluate the performance of the HEMFCs. The operating temperature was set as 95 °C. The H_2_ and O_2_ were humidified at 92 °C and 95 °C, respectively. The flow rates for both H_2_ and O_2_ were 1000 sccm. The back-pressure was set at 250 kPa.

### DFT calculations

First-principles computations were performed using the projector augmented wave method (PAW) as implemented in the Vienna ab-initio simulation package (VASP). The generalized gradient approximation in the form of Perdew-Burke- Ernzerhof (PBE) for the exchange-correction potential and a cutoff energy of 500 eV for the plane-wave basis were adopted. For all calculations, spin polarization was adopted. To consider long-range van der Waals (vdW) interactions, Grimme’s DFT-D3 method was employed. Atomic structures were fully released with converging tolerance for forces on all atoms less than 0.02 eV Å^−1^, and the energy convergence criterion was set to 10^-5 ^eV. 2×2, 5×5, 2×3, 2×3 and 3×2 unit cells of Ir (111), Ir_SA_-N_4_-C, Ir_SA_-Mo_2_C,Ir_NC_-Mo_2_C and IrO_2_ (110) with 4 layers thickness was employed with 15 Å vacuum space in the vacuum-direction to avoid image interactions. The bottom two layers were fixed while the top two layers were relaxed during geometry optimization. 3×3×1, 2×1×2, 3×3×1, 3×3×1 and 3×2×1 Monkhorst–Pack k-meshes were used to sample the Brillouin zone for geometry relaxation. The computational hydrogen electrode model proposed by Nørskov et al.^55^. Was used to evaluate the activity of the catalyst. The Gibbs free energy change, Δ*G*, of each elementary step was obtained based on the following equation, in which the contributions of zero-point energy (Δ*ZPE*) and entropy (*T*Δ*S*) changes were considered:

5 $$\varDelta G=\varDelta E+\varDelta {ZPE}{{{{{\rm{\hbox{-}}}}}}}T\varDelta S-{eU}$$

Where Δ*E* was the calculated energy change of the intermediates, such as *H, *OH and *H_2_O, *U* was the applied electrode potential.

### Supplementary information

Supplementary Information

Peer Review File

### Source data

Source Data

## Data Availability

The data generated in this study are provided in the Source Data file. Source data are provided with this paper.
