# Supplementary material for: Atomically dispersed Iridium on Mo2C as an efficient and stable alkaline hydrogen oxidation reaction catalyst
Source: Nat Commun. 2024 May 18;15:4236. doi: 10.1038/s41467-024-48672-9 (PMC11102501; doi:10.1038/s41467-024-48672-9)
Supplement: Supplementary file 1 — Supplementary Information [file 41467_2024_48672_MOESM1_ESM.pdf]

Supplementary Information for

Atomically dispersed Iridium on Mo_2C as an efficient and stable alkaline hydrogen oxidation reaction catalyst

Jinjie Fang[1,4], Haiyong Wang[1,4], Qian Dang[1,4], Hao Wang[1], Xingdong Wang[1], Jiajing Pei[1], Zhiyuan Xu[1], Chengjin Chen[1], Wei Zhu[1], Hui Li[1,*], Yushan Yan[2,*] and Zhongbin Zhuang[1,3,*]

1. State Key Lab of Organic–Inorganic Composites and Beijing Advanced Innovation Center for Soft Matter Science and Engineering, Beijing University of Chemical Technology, Beijing 100029, China.
2. Department of Chemical and Biomolecular Engineering, University of Delaware, Newark, DE 19716, USA
3. Beijing Key Laboratory of Energy Environmental Catalysis, Beijing University of Chemical Technology, Beijing, 100029, China.
4. These authors contributed equally: Jinjie Fang, Haiyong Wang, Qian Dang.

* Corresponding author: hli@mail.buct.edu.cn, yanys@udel.edu, zhuangzb@mail.buct.edu.cn

Table of contents

Supplementary Figures 1-29	Page 3-31
Supplementary Tables 1-7	Page 32-38
Supplementary Reference	Page 39-40

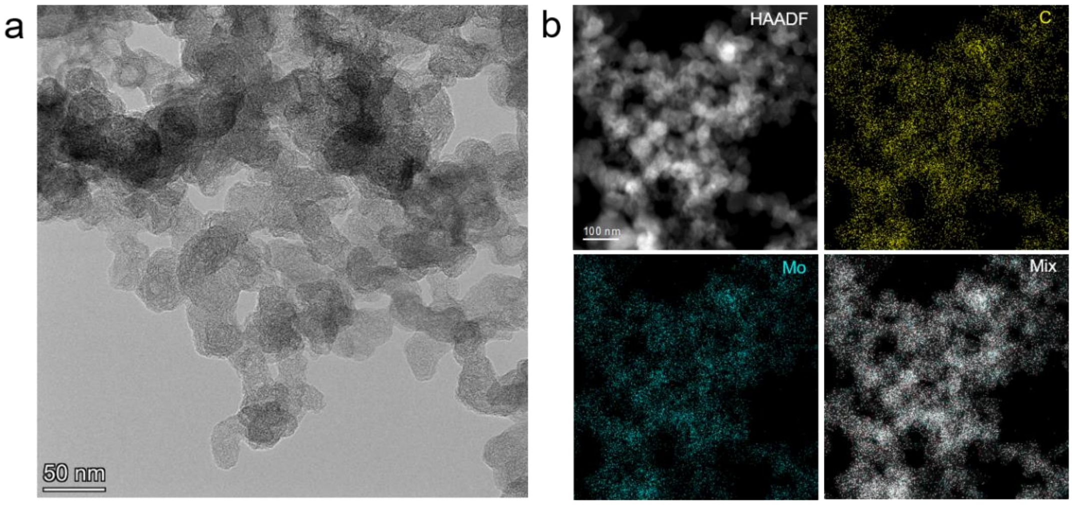

Supplementary Figure 1 | Morphology characterization of MoO_x/C. a, TEM image. **b**, EDS-mapping.

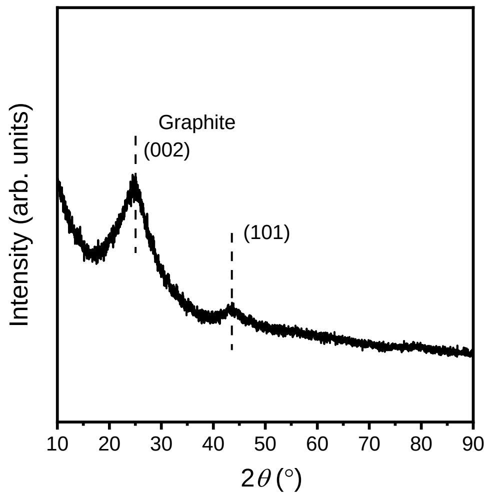

Supplementary Figure 2 | XRD pattern of MoO_x/C.

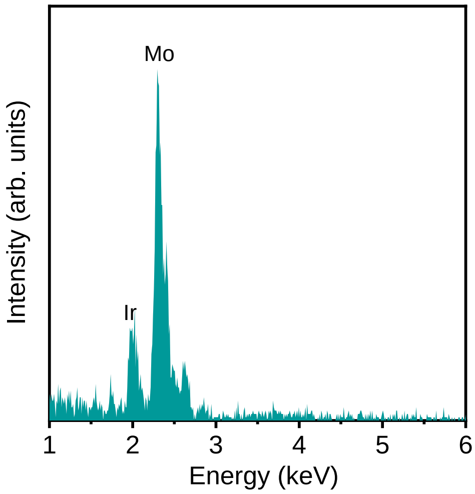

Supplementary Figure 3 | EDS spectra of Ir_{SA}-Mo_2C/C.

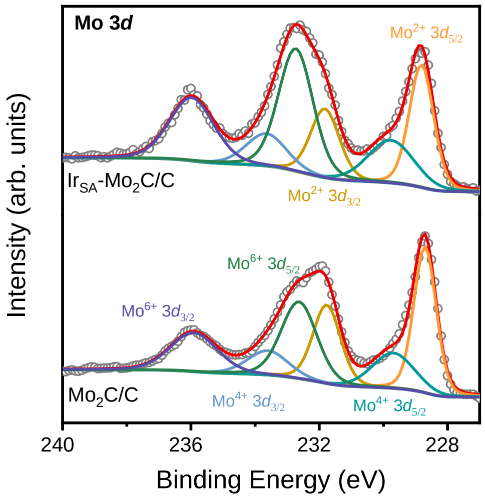

Supplementary Figure 4 | Mo 3*d* XPS deconvolution results of Ir_{SA}-Mo_2C/C and Mo_2C/C.

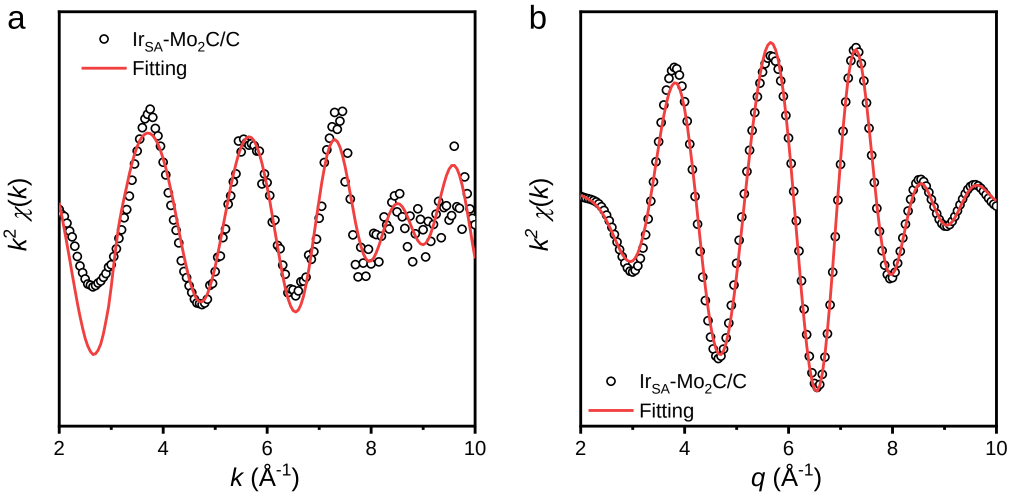

Supplementary Figure 5 | FT-EXAFS fitting curves of Ir L_3-edge of Ir_{SA}-Mo_2C/C. **a**, *k*-space. **b**, *q*-space.

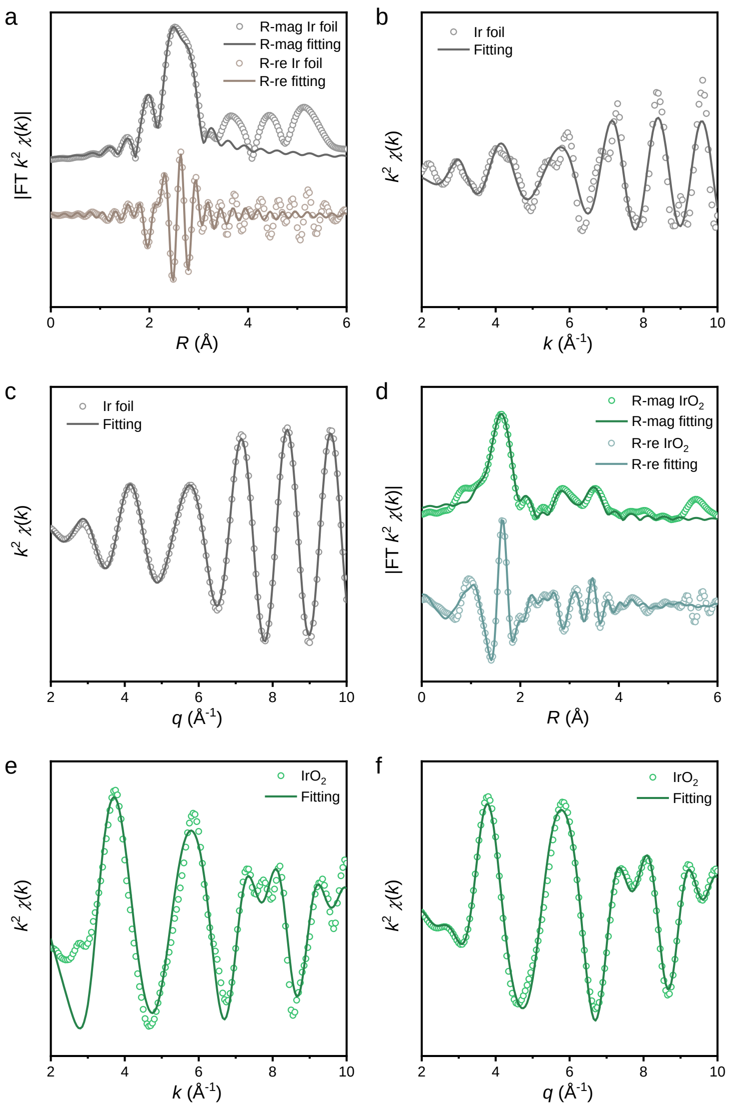

Supplementary Figure 6 | FT-EXAFS fitting results. **a**, Curves of Ir foil at *R*-space. **b**, Curves of Ir foil at *k*-space. **c**, Curves of Ir foil at *q*-space. **d**, Curves of IrO_2 at *R*-space. **e**, Curves of IrO_2 at *k*-space. **f**, Curves of IrO_2 at *q*-space.

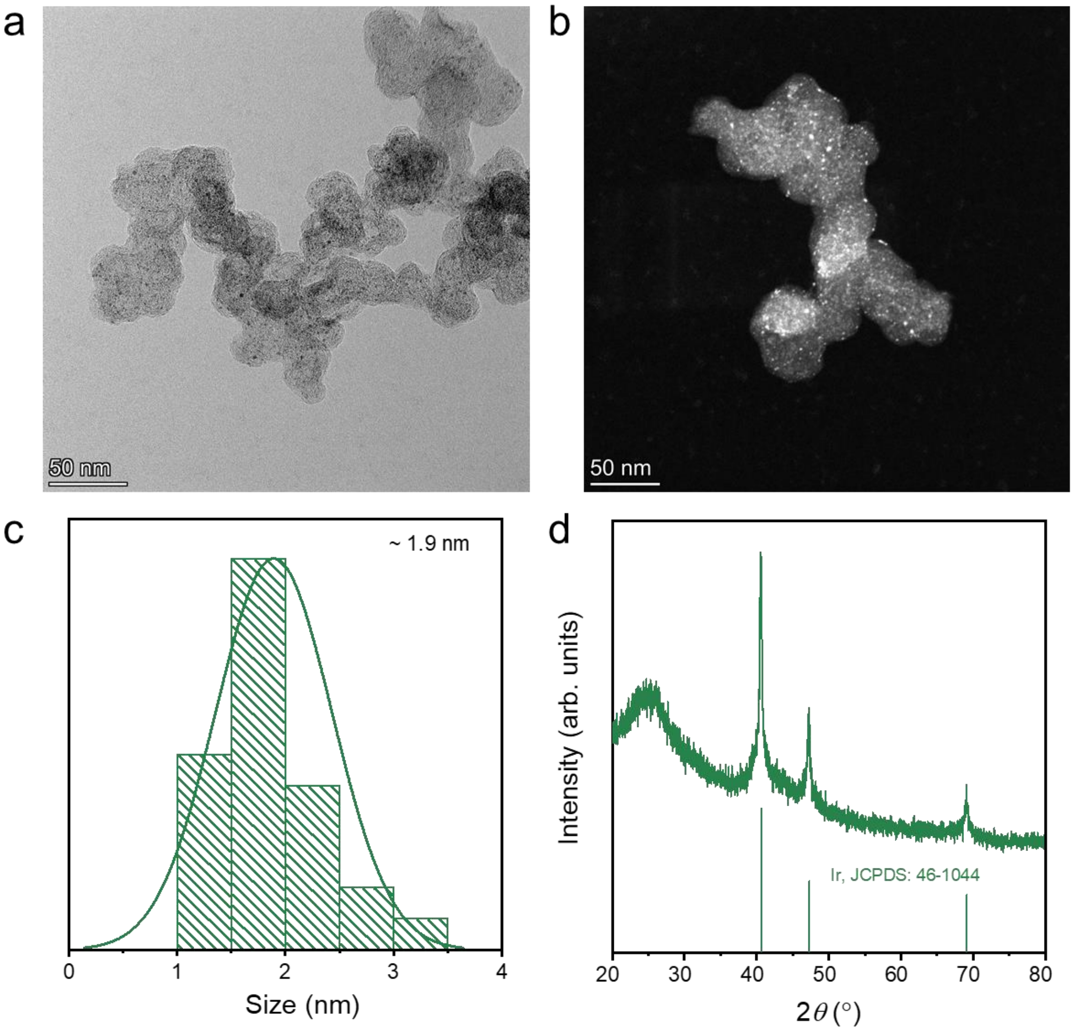

Supplementary Figure 7 | Characterizations of Ir/C. a, TEM image. **b**, HAADF-STEM image. **c**, The size distribution of Ir nanoparticles. **d**, PXRD pattern.

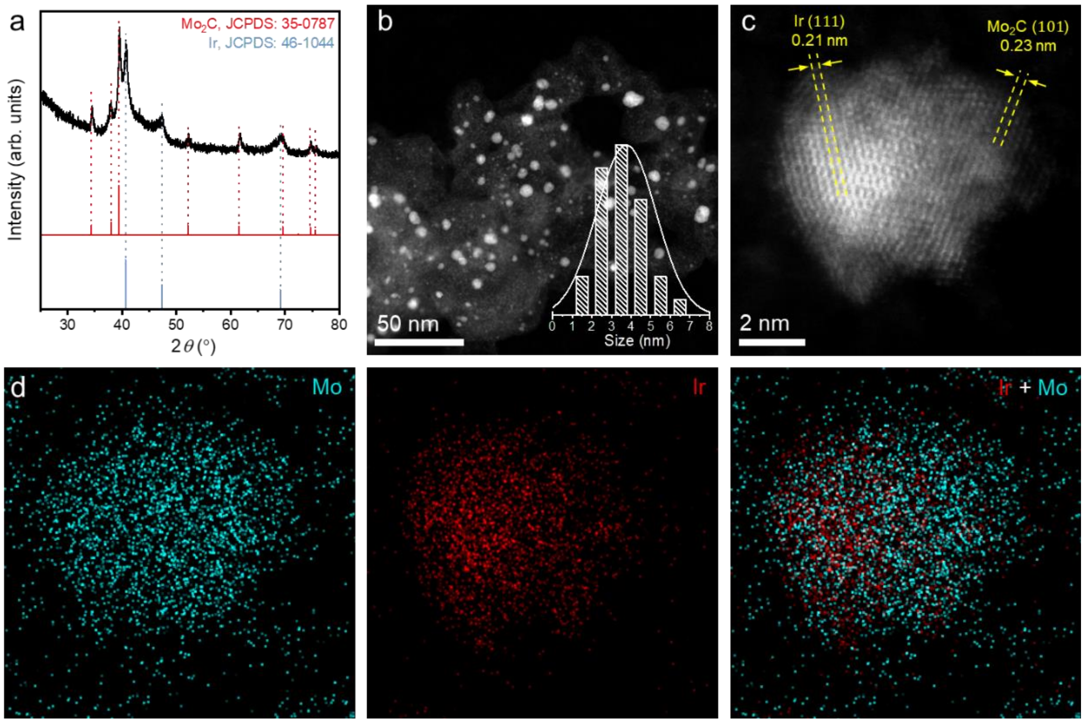

Supplementary Figure 8 | Characterizations of Ir_{NP}-Mo_2C/C. a, PXRD pattern. **b-c**, HAADF-STEM images. **d**, EDS-mapping results of the nanoparticle in c.

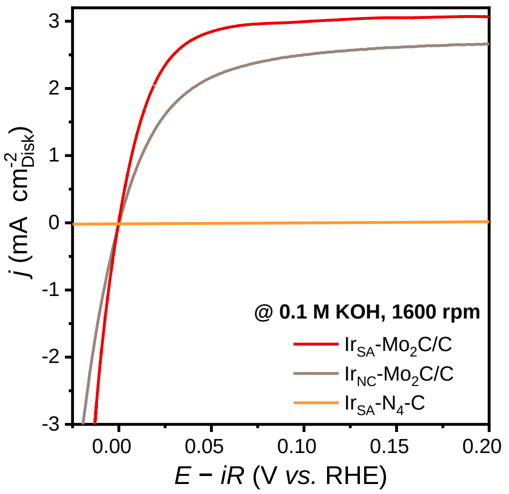

Supplementary Figure 9 | HOR polarization curve of Ir$_{SA}$-N$_4$-C and Ir$_{NC}$-Mo$_2$C/C. The measurement was carried out in H_2-saturated 0.1 M KOH solution with the rotation rate of 1600 rpm and the scan rate of 5 mV s^{-1}. The catalyst loading was ca. 3.2 μg$_{Ir}$ cm^{-2}. The potentials were *iR* corrected and the *R* values for Ir$_{SA}$-Mo$_2$C/C, Ir$_{NC}$-Mo$_2$C/C and Ir$_{SA}$-N$_4$-C measurements was 36.1 ± 0.10, 36.0 ± 0.11 and 35.8 ± 0.07 Ω, respectively.

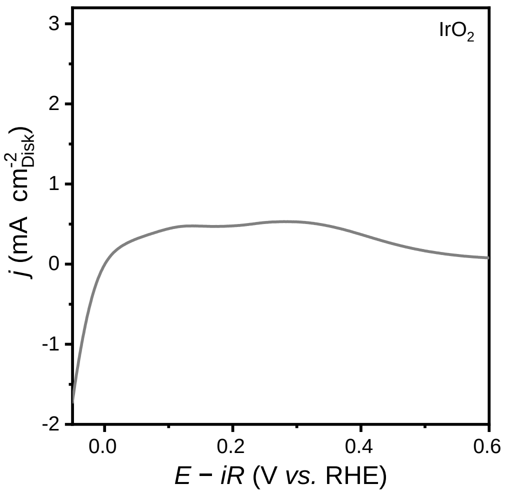

Supplementary Figure 10 | HOR polarization curve of IrO_2. The measurement was carried out in H_2-saturated 0.1 M KOH solution with the rotation rate of 1600 rpm and the scan rate of 5 mV s^{-1}. The catalyst loading was ca. 3.2 μg_{Ir} cm^{-2}. The potentials were *iR* corrected and the *R* value was 36.3 ± 0.10 Ω.

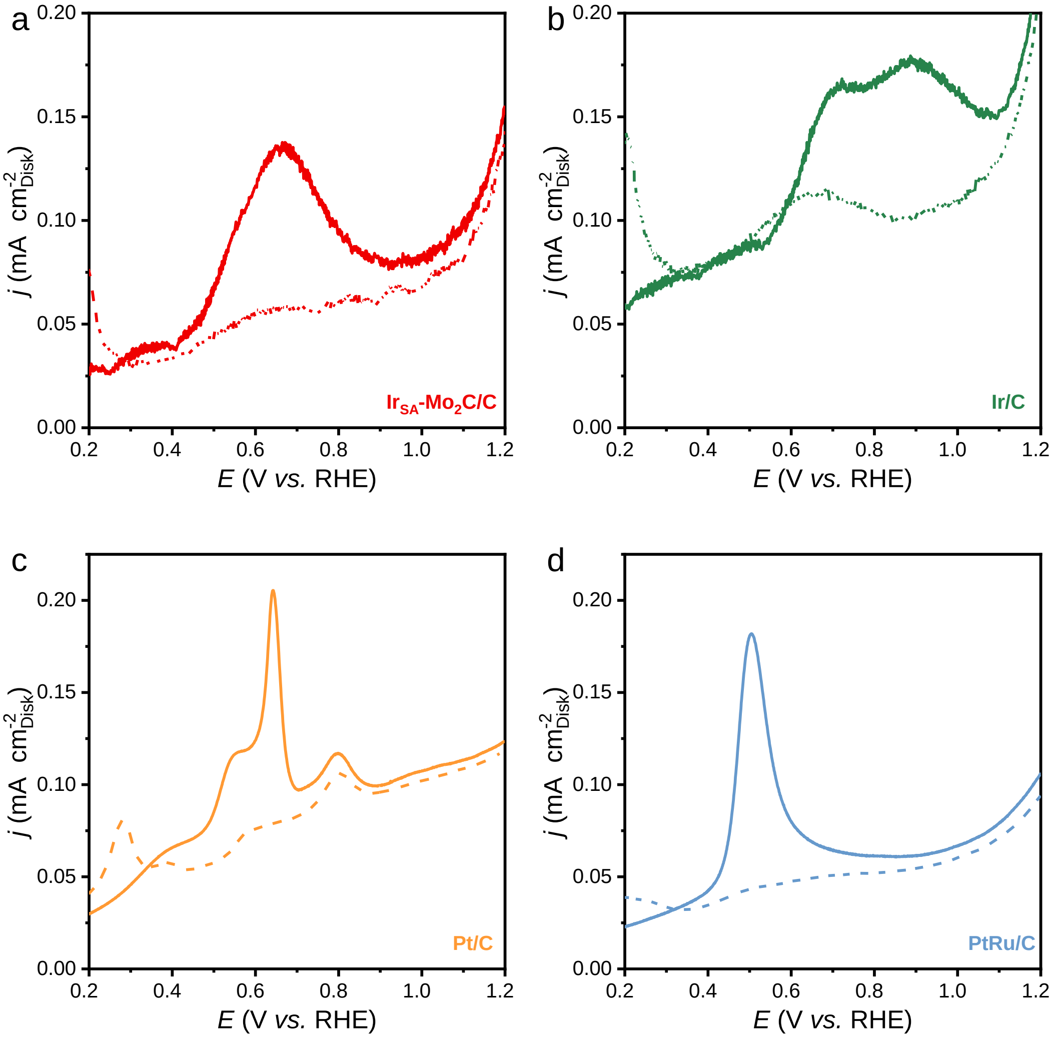

Supplementary Figure 11 | CO-stripping voltammetry. **a**, Ir_{SA}-Mo_2C/C. **b**, Ir/C. **c**, Pt/C. **d**, PtRu/C. The curves were obtained in 0.1 M KOH with the scanning rate of 10 mV s^{-1}. The potentials were displayed without *iR*-correction. The solid line is the CO-stripping voltammetry. The dash lines are CV curves after the CO-stripping.

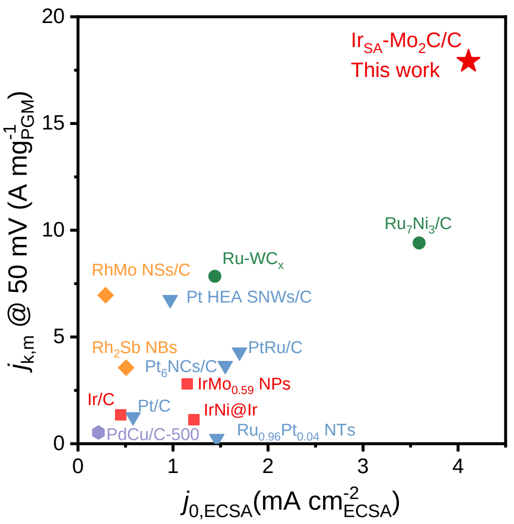

Supplementary Figure 12 | Comparation of the HOR activity. Summary of the $j_{0,ECSA}$ and $j_{k,m}$ at 50 mV of Ir_{SA}-Mo_2C/C and the reported catalysts.

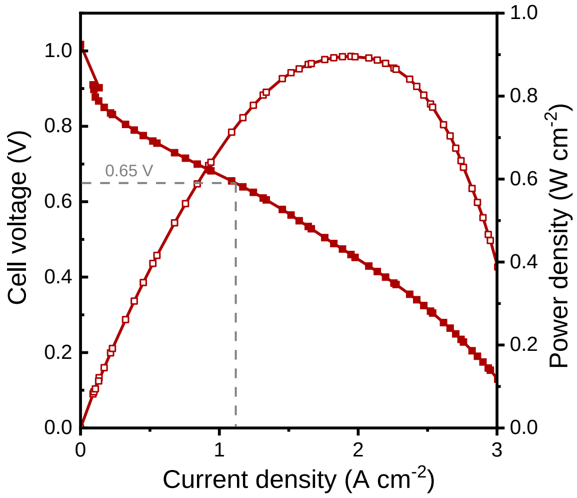

Supplementary Figure 13 | H_2/air (CO_2-free) HEMFC performances based on Ir_{SA}-Mo_2C/C anode catalyst. The cell, anode and cathode humidifier temperatures were 95, 92 and 95 °C, respectively. The anode and cathode were flowed with 1.0 L min^{-1} of H_2 and 1.5 L min^{-1} of air (CO_2-free), respectively. The backpressures were 250 kPag for both sides.

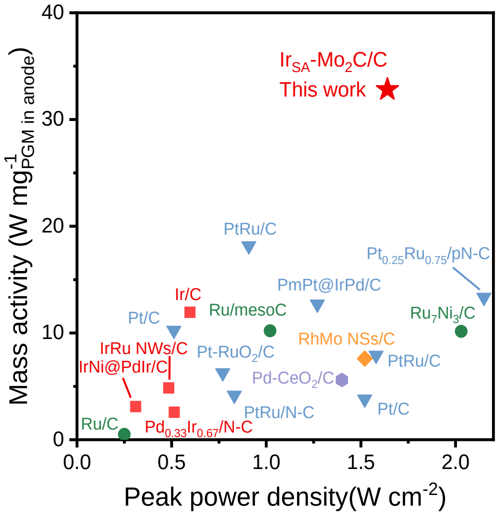

Supplementary Figure 14 | Comparation of the HEMFC performance. Summary of the peak power density and anode PGM based mass activity at peak power of Ir_{SA}-Mo_2C/C and the reported catalysts. The details are listed in Supplementary Table 4.

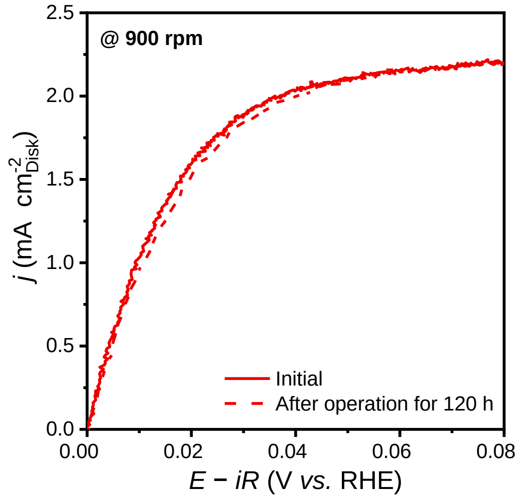

Supplementary Figure 15 | HOR polarization curves in stability test. HOR polarization curves of Ir_{SA}-Mo_2C/C before and after the 120 h of chronoamperpmentry test. The rotation rate was 900 rpm. The scanning rate was 2 mV s^{-1}. The potentials were *iR* corrected and the *R* value was 38.1 ± 0.05 Ω.

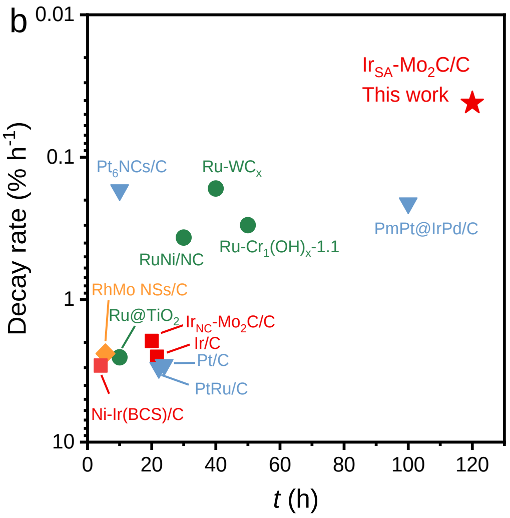

Supplementary Figure 16 | Comparison of the stability of Ir_{SA}-Mo_2C/C with the reported catalysts. The details are listed in Supplementary Table 5.

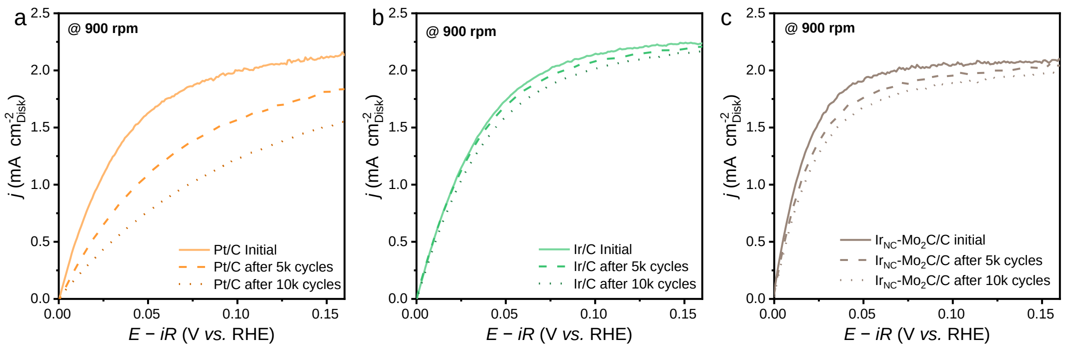

Supplementary Figure 17 | HOR polarization curves in ADT. a, Pt/C. **b**, Ir/C. **c**, Ir_{NC}-Mo_2C/C. The scan rate was 2 mV s^{-1} and the rotation rate was 900 rpm. The potentials were *iR* corrected and the *R* values for Pt/C, Ir/C and Ir_{NC}-Mo_2C/C measurements were 38.3 ± 0.07, 38.2 ± 0.21 and 39.0 ± 0.13 Ω, respectively.

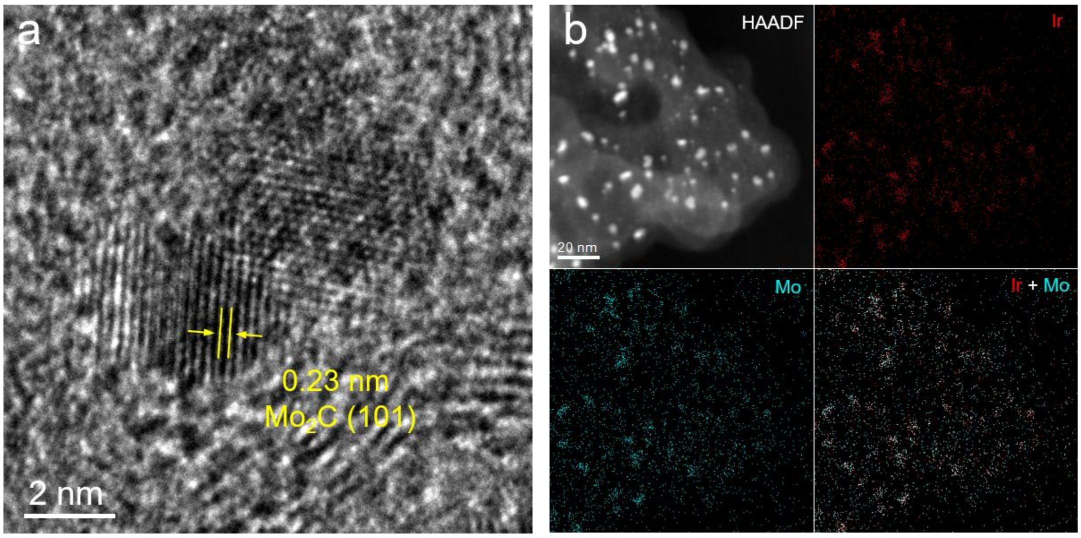

Supplementary Figure 18 | Characterization of Ir_{SA}-Mo_2C/C after ADT. a, HRTEM image. **b**, EDS mappings.

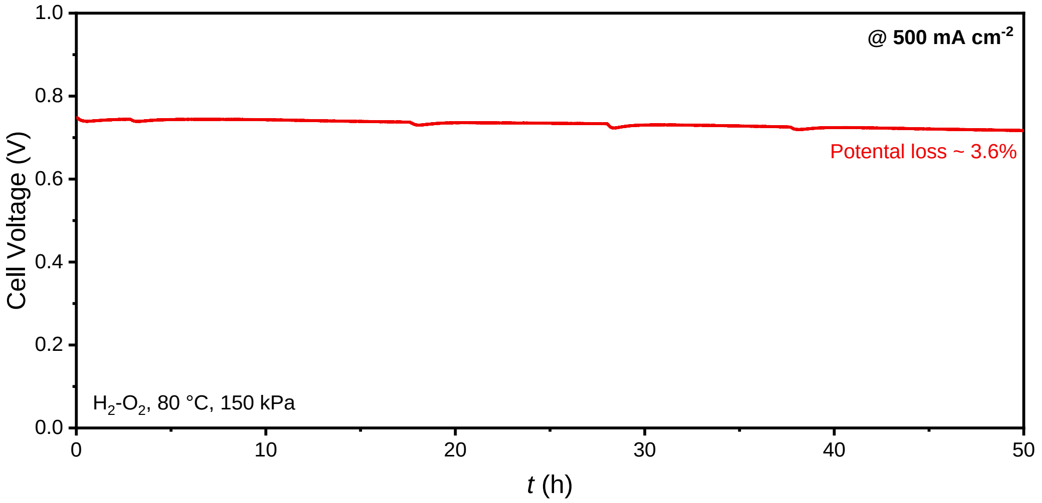

Supplementary Figure 19 | H_2/O_2 HEMFC durability test. The cell was operated at a constant current density of 0.5 A cm^{-2} with Ir_{SA}-Mo_2C/C (0.05 mg_{Ir} cm^{-2}) in anode and Pt/C (0.6 mg_{Pt} cm^{-2}) in cathode. Test conditions: cell temperature of 80 °C, anode humidifier temperature of 78 °C and cathode humidifier temperature of 80 °C, H_2 flow rate at 0.5 L min^{-1} and O_2 flow rate at 0.3 L min^{-1}, backpressures symmetric at 150 kPag.

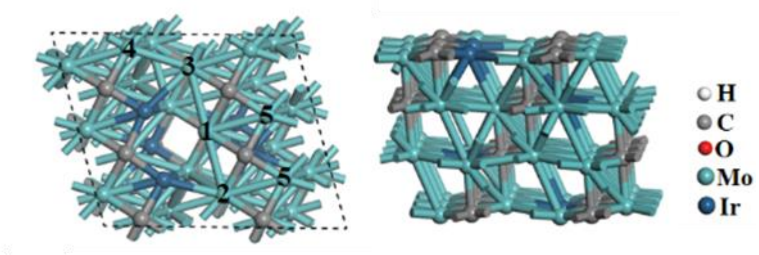

Supplementary Figure 20 | The atomic structure of Ir_{SA}-Mo_2C.

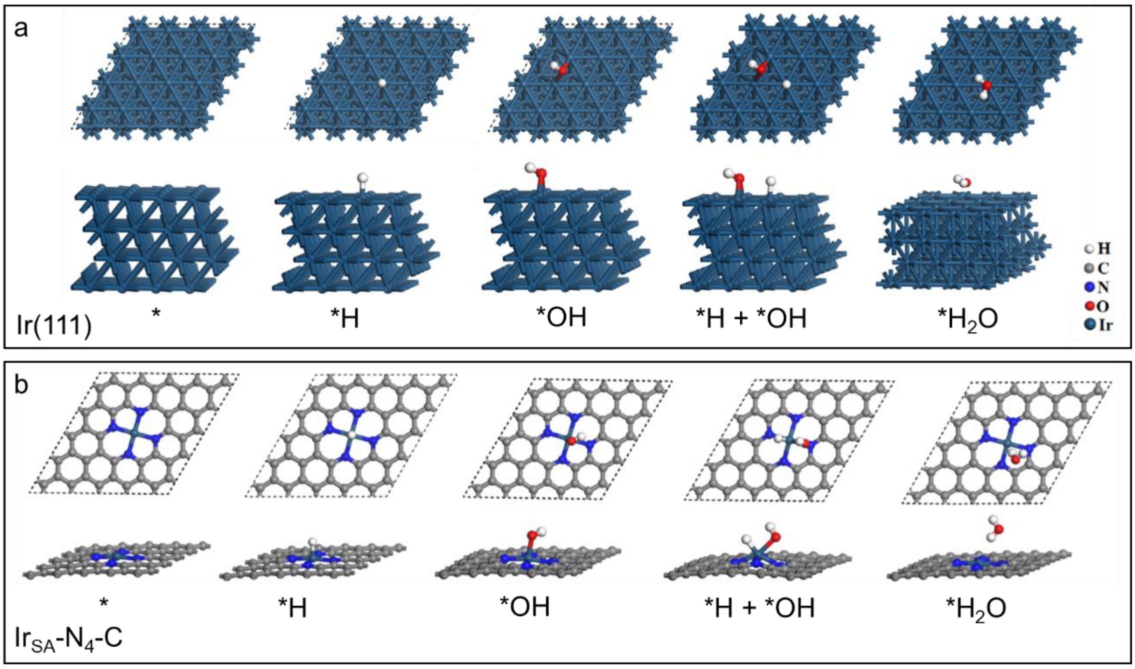

Supplementary Figure 21 | The atomic structure. a, Ir (111) with different HOR intermediates. **b**, Ir_{SA}-N_4-C with different HOR intermediates.

`

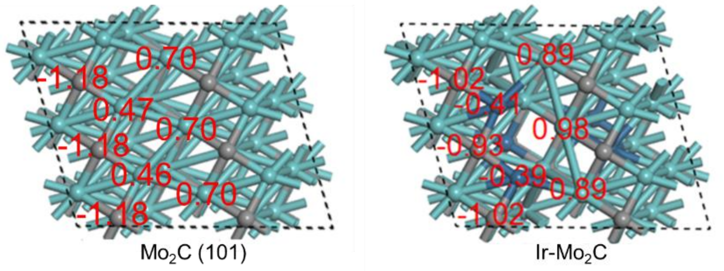

Supplementary Figure 22 | Bader charge of the surface atoms of Mo_2C (101) and Ir_{SA}-Mo_2C.

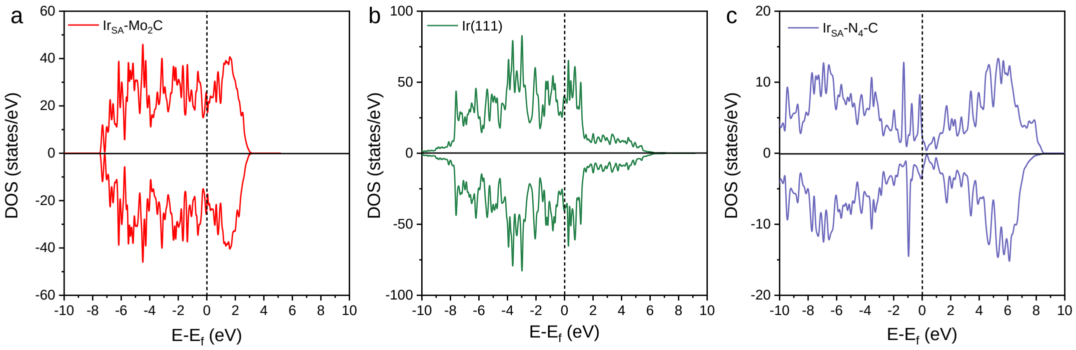

Supplementary Figure 23 | The DOS diagrams. **a**, Ir_{SA}-Mo_2C (101). **b**, Ir (111). **c**, Ir_{SA}-N_4-C.

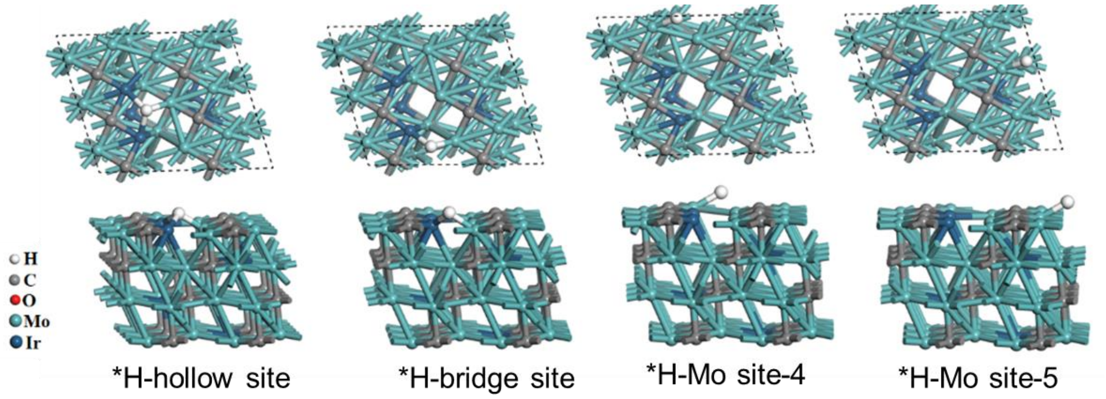

Supplementary Figure 24 | The atomic structure of Ir_{SA}-Mo_2C with adsorbed H on different sites.

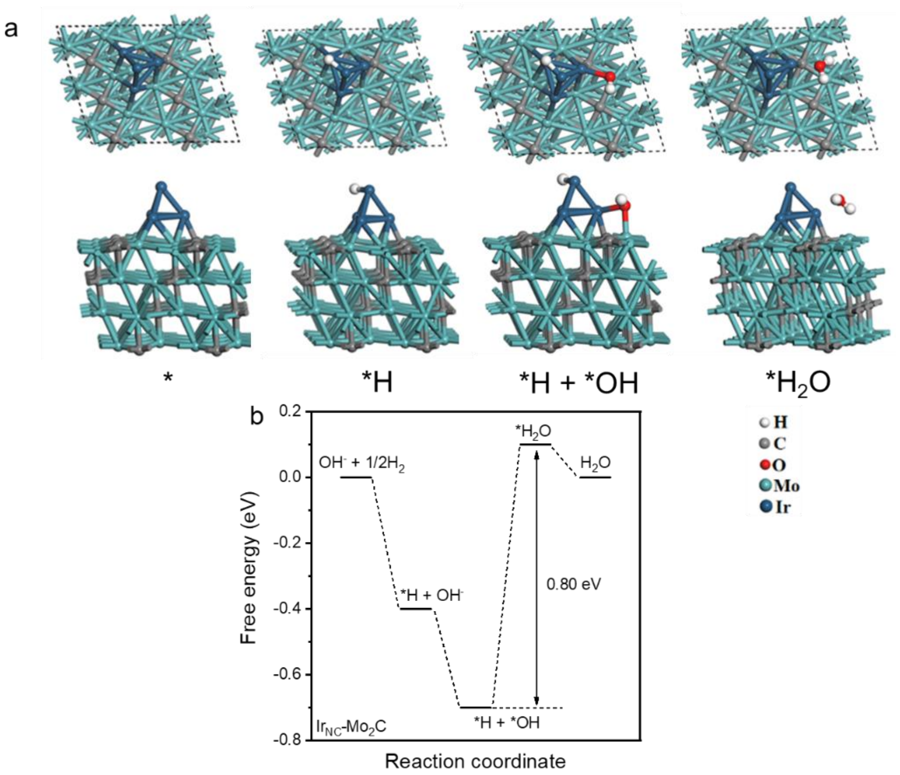

Supplementary Figure 25 | Calculation results of Ir_{NC}-Mo_2C. **a**, The atomic structure of Ir_{NC}-Mo_2C with different HOR intermediates. **b**, The corresponding Gibbs free energy diagram.

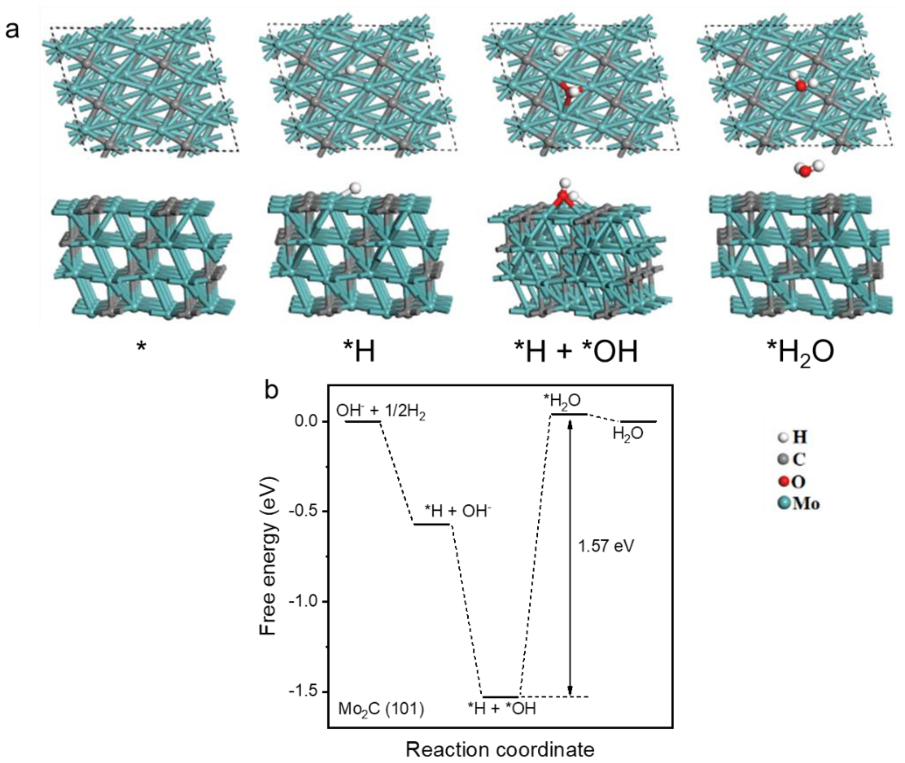

Supplementary Figure 26 | Calculation results of Mo_2C (101). a, The atomic structure of Mo_2C (101) with different HOR intermediates. **b**, The corresponding Gibbs free energy diagram.

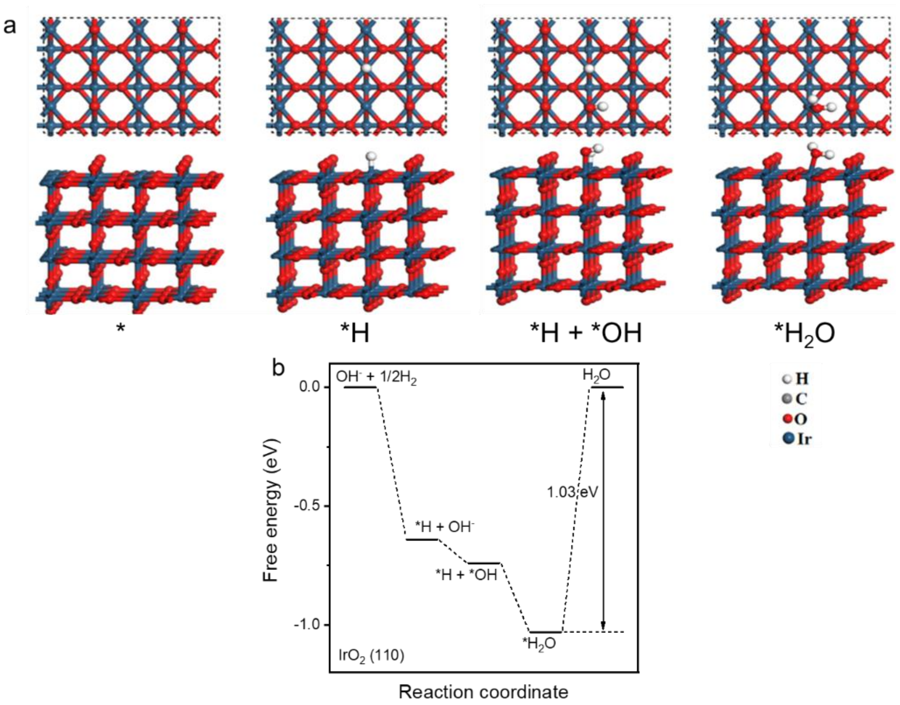

Supplementary Figure 27 | Calculation results of IrO_2 (110). a, The atomic structure of IrO_2 (110) with different HOR intermediates. **b**, The corresponding Gibbs free energy diagram.

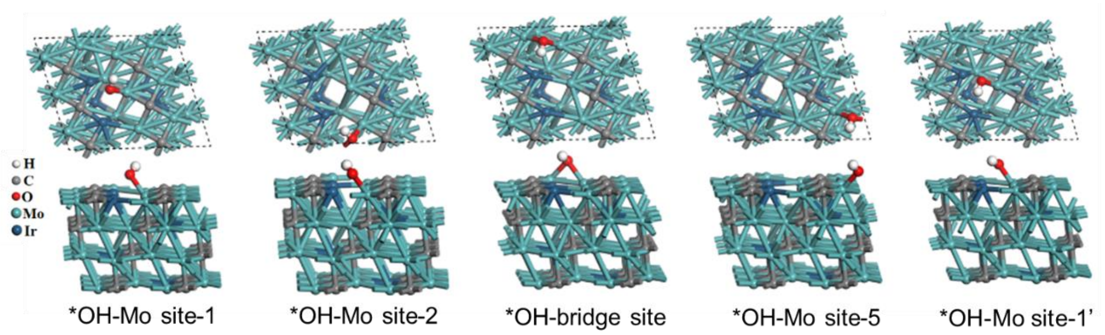

Supplementary Figure 28 | The atomic structure of Ir_{SA}-Mo_2C with adsorbed OH on different sites.

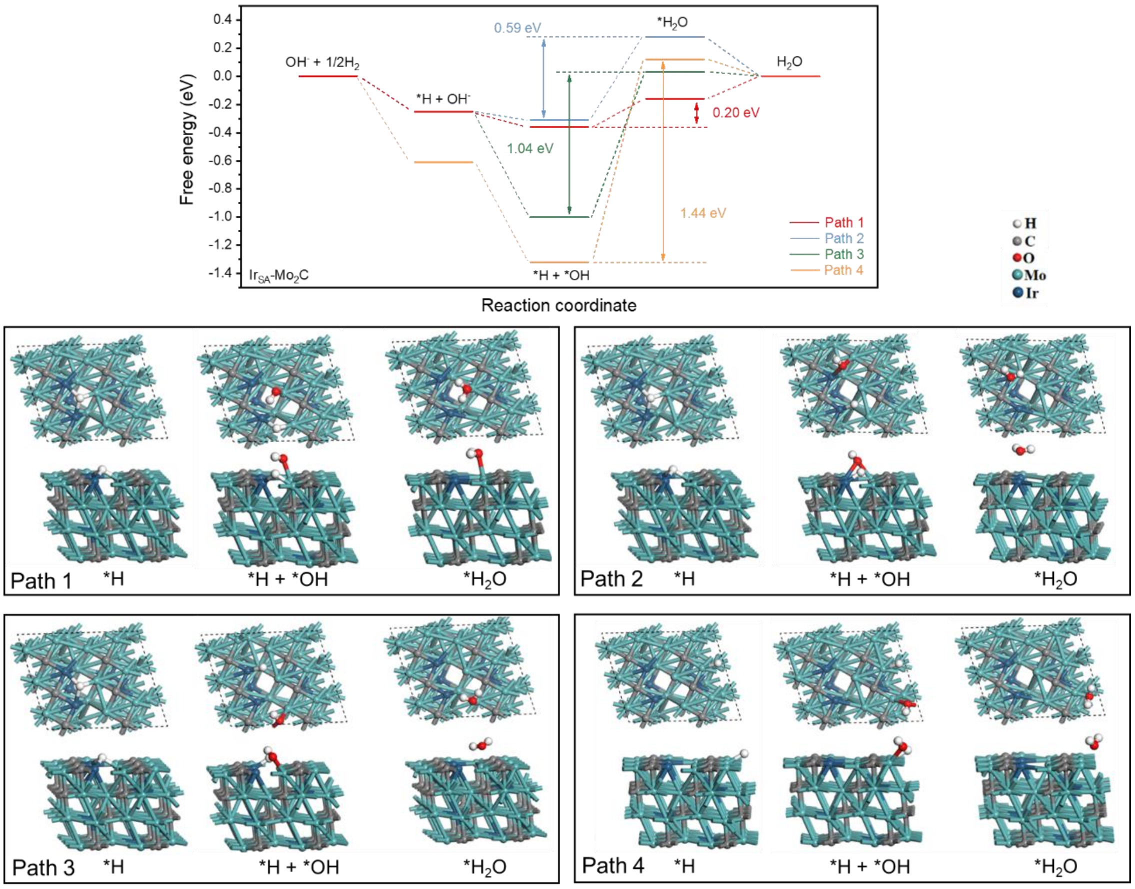

Supplementary Figure 29 | The Gibbs free energy diagrams of HOR on the Ir_{SA}-Mo_2C through four different paths and the corresponding atomic structures of the intermediates.

Supplementary Table 1. Structure parameters extracted from the Ir L_3-edge EXAFS curves fitting for Ir_{SA}-Mo_2C/C.

Sample	Scattering pair	R(Å)[a]	N[b]	σ^2 (10^{-3}Å^2)[c]	ΔE_0 (eV)[d]	r factor
Ir_{SA}-Mo_2C/C	Ir-C	2.10	3.12	3.89	11.5	0.008
	Ir-Mo	2.70	1.40	0.52		
Ir foil	Ir-Ir	2.72	12.0	2.41	9.64	0.005
IrO_2	Ir-O	1.98	5.86	3.49	12.5	0.016
	Ir-O-Ir	3.16	6.19	8.56		

[a]R: Bond distance; [b]N: coordination numbers; [c]σ^2: Debye-Waller factors; [d]ΔE_0: the inner potential correction. r factor: goodness of fit.

Supplementary Table 2. The catalyst loading, ECSA and ECSA normalized j_0 of Ir_{SA}-Mo_2C/C, Ir/C, PtRu/C and Pt/C.

Catalyst	Loading (μg_{PGM} cm^{-2})	ECSA (m^2 g_{PGM}^{-1})	i_0 (mA)	$j_{0,ECSA}$ (mA cm^{-2}_{ECSA})
Ir_{SA}-Mo_2C/C	3.2	156	3.99	4.1
Ir/C	3.2	128	0.368	0.46
PtRu/C	3.2	107	1.79	1.7
Pt/C	3.2	97	0.353	0.58

Supplementary Table 3. Summary of the alkaline HOR activity tested by RDE method.

Type	Catalyst	loading (mg_{PGM} cm^{-2})	$j_{0,ECSA}$ (mA cm^{-2}_{ECSA})	$j_{k,m}$ at 50 mV (A mg_{PGM}^{-1})	Reference
Ir	Ir_{SA}-Mo_2C/C	3.2	4.1	18.0	This work
	Ir/C	3.2	0.45	1.35	This work
	IrNi@Ir	10	1.22	1.12	[1]
	Ir/MoS_2	20	1.28	\	[2]
	Ir/O-MoO_2	25	1.96	\	[3]
	Ir_1Ru_9/C	3.5	0.90	\	[4]
	$IrMo_{0.59}$ NPs	10	1.15	2.80	[5]
	Ir_1-P_1/NPG	4.3	\	1.71	[6]
	Ni-Ir(BCS)/G	32	\	0.33	[7]
Pt	Pt_6NCs/C	5.0	1.55	3.66	[8]
	La_1Pt@HCS	10	1.55	\	[9]
	$Ru_{0.96}Pt_{0.04}$ NTs	7.0	1.46	0.24	[10]
	Pt HEA SNWs/C	8.8	0.97	6.75	[11]
	$Pt_{0.25}Ru_{0.75}$/pN-C	2.3	2.12	\	[12]
	PtRu/C	3.2	1.7	4.29	This work
	Pt/C	3.2	0.58	1.26	This work
Other PGM	Ru_7Ni_3/C	3.9	3.59	9.40	[13]
	Ru-WC_x	1.2	1.44	7.84	[14]
	RhMo NSs/C	18	0.29	6.96	[15]
	Pd-Pd_4S/C	42	0.23	\	[16]
	Rh_2Sb NBs	6.4	0.51	3.56	[17]
	Ru-$Cr_1(OH)_x$-1.1	60	\	0.43	[18]
	PdCu/C-500℃	13	0.22	0.52	[19]

Supplementary Table 4. Summary of the HEMFC performances with different anode catalysts.

Anode (mg_{PGM} cm^{-2})	Cathode (mg_{PGM} cm^{-2})	T_{cell} (°C)	Back-pressure	P_{max} (W cm^{-2})	*i* @ 0.65V (A cm^{-2})	Mass activity (W mg_{PGM}^{-1})	Reference
Ir-based anode							
Ir_{SA}-Mo_2C/C (0.05)	Pt/C (0.4)	95	250	1.64	1.60	32.8	This work
Ir/C (0.05)	Pt/C (0.4)	95	250	0.60	0.43	11.9	This work
IrRu NWs/C (0.1)	Pt/C (0.3)	60	100	0.485	0.500	4.85	[20]
IrNi@PdIr/C (0.2)	Pt/C (0.3)	60	0	0.311	0.300	1.55	[21]
$Pd_{0.33}Ir_{0.67}$/N-C (0.2)	Pt/C (0.3)	79	100	0.514	0.605	2.57	[22]
Pt-based anode							
PtRu/C (0.05)	Pt/C (0.4)	95	250	0.91	0.76	18.2	This work
Pt/C(0.05)	Pt/C (0.4)	95	250	0.51	0.49	10.2	This work
PtRu/C (0.2)	Pt/C (0.4)	95	250	1.58	1.68	7.90	[13]
Pt/C (0.4)	Pt/C (0.4)	95.5	250	1.52	1.54	3.80	[23]
PmPt@IrPd/C (0.1)	Pt/C (0.4)	80	200	1.27	1.38	12.7	[24]
Pt-RuO_2/C (0.123)	Pt/C (0.6)	80	285	0.770	1.02	6.26	[25]
PtRu/N-C (0.2)	Pt/C (0.4)	80	0	0.831	0.990	4.16	[26]
$Pt_{0.25}Ru_{0.75}$/pN-C (0.18)	Pt/C (0.55)	80	0	2.15	2.40	13.3	[12]
Other PGM-based anode							
Ru_7Ni_3/C (0.2)	Pt/C (0.4)	95	250	2.03	2.00	10.2	[13]
Ru/mesoC (0.1)	Pt/C (0.45)	80	100	1.02	1.05	10.2	[27]
Ru/C (0.5)	Pt/C (0.5)	70	0	0.250	0.200	0.50	[28]
RhMo NSs (0.2)	Pt/C (0.2)	80	200	1.52	1.36	7.60	[15]
Pd-CeO_2/C (0.25)	Pt/C (0.4)	80	0	1.40	1.40	5.60	[29]

Supplementary Table 5. Summary of the alkaline HOR stability of the alkaline HOR catalysts.

Type	Catalyst	Loading (mg_{PGM} cm^{-2})	Stability (retain, time)	Decay rate (% h^{-1})	Overpotential (mV)	Method	Reference
Ir	Ir_{SA}-Mo_2C/C	7.0	95.0%, 120h	0.042	50	RDE	This work
	Ir_{NC}-Mo_2C/C	7.0	61.5%, 20h	1.9	50	RDE	This work
	Ir/C	7.0	45.5%, 21.7h	2.5	50	RDE	This work
	Ni-Ir(BCS)/G	32	88.1%, 4.10h	2.90	100	RDE	[7]
Pt	PtRu/C	7.0	33.2%, 22.2h	3.0	50	RDE	This work
	Pt/C	7.0	31.5%, 23.9h	2.9	50	RDE	This work
	Pt_6NCs/C	5.0	98.3%, 1.7h	0.170	60	RDE	[8]
	PmPt@IrPd/C	5.0	79.0%, 100h	0.210	50	GDE	[24]
Other PGM	RuNi/NC	25	89.0%, 30h	0.367	50	RDE	[30]
	Ru@TiO_2	25	74.4%, 10h	2.54	100	GDE	[31]
	Ru-WC_x	1.2	93.4%, 40h	0.166	100	RDE	[14]
	RhMo NSs/C	18	86.7@, 5.56h	2.39	100	RDE	[15]
	Ru-$Cr_1(OH)_x$-1.1	2000	85.0%, 50h	0.300	50	GDE	[18]

Supplementary Table 6. Cell parameters of Ir, Graphene, IrO_2, Mo_2C, Ir_{SA}-Mo_2C and Ir_{NC}-Mo_2C.

	a (Å)	*b* (Å)	*c* (Å)	*α* (°)	*β* (°)	*γ* (°)
Ir	3.84	3.84	3.84	90	90	90
IrO_2	4.53	4.53	3.19	90	90	90
Mo_2C	3.05	3.05	4.62	90	90	120

Supplementary Table 7. Gibbs free energy of H and OH on different active sites of Ir_{SA}-Mo_2C/C.

Initial site	H adsorption		OH adsorption	
	Final site	ΔG_H (eV)	Final site	ΔG_{OH} (eV)
Mo site-1	hollow site	−0.25	Mo site-1	0.01
Mo site-2	bridge site	−0.41	Mo site-2	−0.60
Mo site-3	bridge site	−0.41	bridge site	−0.61
Mo site-4	Mo site-4	−0.50	bridge site	-0.61
Mo site-5	Mo site-5	−0.61	Mo site-5	−0.70
Ir site	bridge site	−0.41	Mo site-1’	−0.05

Supplementary reference

1 Liu, D. et al. One-pot synthesis of IrNi@Ir core-shell nanoparticles as highly active hydrogen oxidation reaction electrocatalyst in alkaline electrolyte. *Nano Energy* **59**, 26-32 (2019).

2 Liu, D.-Q. et al. Tailoring Interfacial Charge Transfer of Epitaxially Grown Ir Clusters for Boosting Hydrogen Oxidation Reaction. *Adv. Energy Mater.* **13**, 2202913 (2023).

3 Li, M. et al. Revealing the Regulation Mechanism of Ir–MoO_2 Interfacial Chemical Bonding for Improving Hydrogen Oxidation Reaction. *ACS Catal.* **11**, 14932-14940 (2021).

4 Wang, H. & Abruña, H. D. IrPdRu/C as H_2 Oxidation Catalysts for Alkaline Fuel Cells. *J. Am. Chem. Soc.* **139**, 6807-6810 (2017).

5 Fu, L. et al. IrMo Nanocatalysts for Efficient Alkaline Hydrogen Electrocatalysis. *ACS Catal.* **10**, 7322-7327 (2020).

6 Wang, Q. et al. Atomic metal-non-metal catalytic pair drives efficient hydrogen oxidation catalysis in fuel cells. *Nat. Catal.* **6**, 916-926 (2023).

7 Tang, T. et al. Unconventional Bilateral Compressive Strained Ni–Ir Interface Synergistically Accelerates Alkaline Hydrogen Oxidation. *J. Am. Chem. Soc.* **145**, 13805-13815 (2023).

8 Wang, X. et al. Atomic-precision Pt_6 nanoclusters for enhanced hydrogen electro-oxidation. *Nat. Commun.* **13**, 1596 (2022).

9 Wang, X. et al. Embedding oxophilic rare-earth single atom in platinum nanoclusters for efficient hydrogen electro-oxidation. *Nat. Commun.* **14**, 3767 (2023).

10 St. John, S. et al. Platinum and Palladium Overlayers Dramatically Enhance the Activity of Ruthenium Nanotubes for Alkaline Hydrogen Oxidation. *ACS Catal.* **5**, 7015-7023 (2015).

11 Zhan, C. et al. Subnanometer high-entropy alloy nanowires enable remarkable hydrogen oxidation catalysis. *Nat. Commun.* **12**, 6261 (2021).

12 Ni, W. et al. Synergistic interactions between PtRu catalyst and nitrogen-doped carbon support boost hydrogen oxidation. *Nat. Catal.* **6**, 773-783 (2023).

13 Xue, Y. et al. A highly-active, stable and low-cost platinum-free anode catalyst based on RuNi for hydroxide exchange membrane fuel cells. *Nat. Commun.* **11**, 5651 (2020).

14 Wang, L. et al. Stabilizing Low-Valence Single Atoms by Constructing Metalloid Tungsten Carbide Supports for Efficient Hydrogen Oxidation and Evolution. *Angew. Chem. Int. Ed.* **62**, e202311937 (2023).

15 Zhang, J. et al. Atomic-thick metastable phase RhMo nanosheets for hydrogen oxidation catalysis. *Nat. Commun.* **14**, 1761 (2023).

16 Su, L. et al. Identifying the Role of Hydroxyl Binding Energy in a Non-Monotonous Behavior of Pd-Pd_4S for Hydrogen Oxidation Reaction. *Adv. Funct. Mater.* **32**, 2113047 (2022).

17 Zhang, Y. et al. Atomically Isolated Rh Sites within Highly Branched Rh_2Sb Nanostructures Enhance Bifunctional Hydrogen Electrocatalysis. *Adv. Mater.* **33**, 2105049 (2021).

18 Zhang, B. et al. Atomically dispersed chromium coordinated with hydroxyl clusters

enabling efficient hydrogen oxidation on ruthenium. *Nat. Commun.* **13**, 5894 (2022).

19 Qiu, Y. et al. BCC-Phased PdCu Alloy as a Highly Active Electrocatalyst for Hydrogen Oxidation in Alkaline Electrolytes. *J. Am. Chem. Soc.* **140**, 16580-16588 (2018).

20 Qin, B. et al. Ultrathin IrRu nanowire networks with high performance and durability for the hydrogen oxidation reaction in alkaline anion exchange membrane fuel cells. *J. Mater. Chem. A* **6**, 20374-20382 (2018).

21 Qin, B. et al. A novel IrNi@PdIr/C core–shell electrocatalyst with enhanced activity and durability for the hydrogen oxidation reaction in alkaline anion exchange membrane fuel cells. *Nanoscale* **10**, 4872-4881 (2018).

22 Cong, Y. et al. Uniform $Pd_{0.33}Ir_{0.67}$ nanoparticles supported on nitrogen-doped carbon with remarkable activity toward the alkaline hydrogen oxidation reaction. *J. Mater. Chem. A* **7**, 3161-3169 (2019).

23 Wang, T. et al. High-Performance Hydroxide Exchange Membrane Fuel Cells through Optimization of Relative Humidity, Backpressure and Catalyst Selection. *J. Electrochem. Soc.* **166**, F3305 (2019).

24 Zhao, T. et al. Pseudo-Pt Monolayer for Robust Hydrogen Oxidation. *J. Am. Chem. Soc.* **145**, 4088-4097 (2023).

25 Wang, R. et al. Ultrafine Pt cluster and RuO_2 heterojunction anode catalysts designed for ultra-low Pt-loading anion exchange membrane fuel cells. *Nanoscale Horiz.* **5**, 316-324 (2020).

26 Cong, Y., Chai, C., Zhao, X., Yi, B. & Song, Y. $Pt_{0.25}Ru_{0.75}$/N-C as Highly Active and Durable Electrocatalysts toward Alkaline Hydrogen Oxidation Reaction. *Adv. Mater. Interfaces* **7**, 2000310 (2020).

27 Zeng, L. et al. Extraordinary activity of mesoporous carbon supported Ru toward the hydrogen oxidation reaction in alkaline media. *J. Power Sources* **461**, 228147 (2020).

28 Ohyama, J., Sato, T. & Satsuma, A. High performance of Ru nanoparticles supported on carbon for anode electrocatalyst of alkaline anion exchange membrane fuel cell. *J. Power Sources* **225**, 311-315 (2013).

29 Bellini, M. et al. Palladium–Ceria Catalysts with Enhanced Alkaline Hydrogen Oxidation Activity for Anion Exchange Membrane Fuel Cells. *ACS Appl. Energy Mater.* **2**, 4999-5008 (2019).

30 Han, L. et al. Design of Ru-Ni diatomic sites for efficient alkaline hydrogen oxidation. *Sci. Adv.* **8**, eabm3779 (2022).

31 Zhou, Y. et al. Lattice-confined Ru clusters with high CO tolerance and activity for the hydrogen oxidation reaction. *Nat. Catal.* **3**, 454-462 (2020).
